# The adjacent ATP-binding protein-encoding genes of the *Enterococcus faecalis* phosphate-specific transport (*pst*) locus have non-overlapping cellular functions

**DOI:** 10.1128/jb.00033-25

**Published:** 2025-04-14

**Authors:** Christopher M. Healy, Evelyn A. Pham, Keane J. Dye, Candace N. Rouchon, Biko McMillan, Kristi L. Frank

**Affiliations:** 1 Department of Microbiology and Immunology, Uniformed Services University of the Health Sciences 1685, Bethesda, Maryland, USA; 2 Henry M. Jackson Foundation for the Advancement of Military Medicine, Inc., Bethesda, Maryland, USA; University of Illinois Chicago, Chicago, Illinois, USA

**Keywords:** inorganic phosphate, commensal, import, ATPase, phosphorus, pathogen, gene expression, bacterial growth, metabolism

## Abstract

The widely conserved *pst-phoU* operon encodes a low-velocity, high-affinity, ATP-dependent importer for inorganic phosphate (Pi). The *pstB* gene encodes the ATPase that powers the import of Pi into the cell. In some Firmicutes, including the gastrointestinal commensal and opportunistic pathogen *Enterococcus faecalis*, the *pst-phoU* locus contains adjacent *pstB* genes. In this work, we compared the functionality of *E. faecalis pstB1* and *pstB2. E. faecalis pstB1* and *pstB2* share sequence similarities with verified PstB ATPases from *Escherichia coli* and *Streptococcus pneumoniae* and only share ~60% amino acid identity with each other. Deletion of *pstB1* was associated with a growth defect in low Pi-containing chemically defined medium (CDM), reduced Pi uptake, and a moderate increase in alkaline phosphatase (AP) activity. Deletion of *pstB2* fully inhibited growth in CDM regardless of inorganic phosphorus source but did not hinder growth in rich, undefined medium. The Δ*pstB2* mutant also exhibited a significant increase in AP activity that was associated with extracellular Pi accumulation. Overexpression of *pstB2* in the *pstB1* mutant was sufficient to restore growth in low-Pi CDM, Pi uptake, and AP activity, but this was not recapitulated with overexpression of *pstB1* in the Δ*pstB2* mutant. Deletion of either *pstB* paralog increased expression of the tandem paralog, and overexpression of *pstB2* in Δ*pstB2* reduced *pstB1* expression. These results suggest that the *E. faecalis pstB2*-encoded ATPase is required for Pi import, while the *pstB1*-encoded ATPase has an accessory role in Pi import that can be duplicated by the presence of excess PstB2.

**IMPORTANCE:**

Phosphate is critical for all microbial life. In many bacteria, inorganic phosphate (Pi) is imported by the high-affinity, low-velocity Pst-PhoU system. The *pstB* gene encodes the ATPase that powers Pi import. The *pst-phoU* operon in many Firmicutes, including the human commensal and opportunistic pathogen *Enterococcus faecalis*, contains adjacent *pstB* genes, *pstB1* and *pstB2*. No studies on the relative biological contributions of tandem *pstB* paralogs in any microbe have been published. This genetic study indicates that *E. faecalis pstB1* and *pstB2* do not have equivalent functions. The *pstB2* gene encodes an ATPase that is required for Pi import, while the ATPase encoded by *pstB1* has an accessory role in Pi import that can be duplicated by the presence of excess PstB2.

## INTRODUCTION

Phosphorus is the 11th most abundant element present in the Earth’s crust and is necessary for the formation of macromolecules that are critical for all terrestrial life (1). Phosphorus is primarily found in its most oxidized form, PO_4_^3-^, which is commonly referred to as the orthophosphate or inorganic phosphate (Pi) anion (2). Unsurprisingly, organisms have evolved multiple mechanisms to take advantage of the abundance of Pi in order to meet cellular needs for phosphorus. One such mechanism that bacteria use to acquire Pi directly from their environment and transport it into the cytoplasm of the cell is the phosphate-specific transport (Pst) system. The Pst system, which is encoded by the *pst-phoU* operon, is a high-affinity, low-velocity Pi importer that is most active when external Pi levels fall below a certain threshold (3, 4). Pst signals through the PhoB (response regulator)-PhoR (histidine kinase) two-component system, and disruption of the *pst* system results in a variety of phenotypic changes across bacterial species. For example, *Cronobacter sakazakii* Δ*pst* mutants exhibited decreased biofilm formation and increased adhesion when grown in low Pi (5). Deletion of the *pst* operon in avian pathogenic *Escherichia coli* resulted in reduced virulence in an infected chicken model (6). In uropathogenic *E. coli*, deletion of *pst* resulted in diminished colonization of the urinary tracts of mice and rendered mutants less invasive in human bladder epithelial cells, likely due to decreased expression of type 1 fimbriae (7).

**Fig 1:**
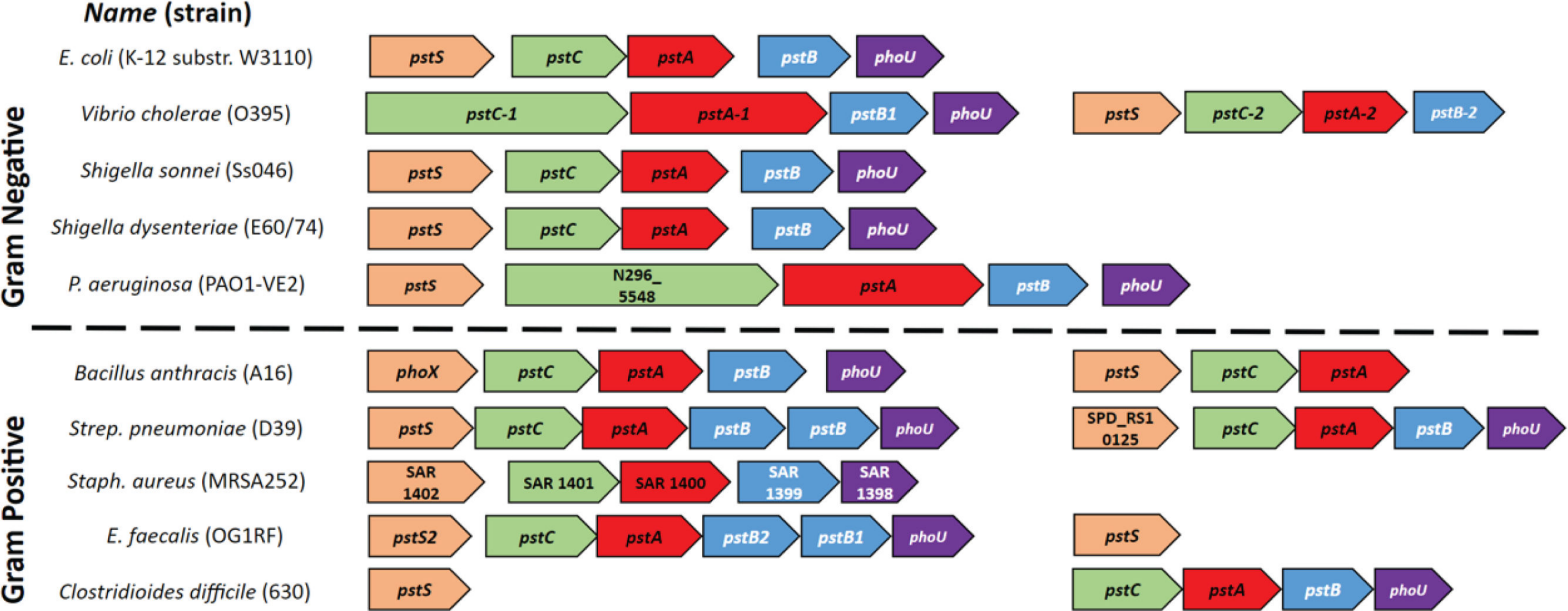
The *pst-phoU* operon is well conserved throughout the bacterial kingdom. The genomic organization of *pst-phoU* loci from selected gram-negative and gram-positive bacterial species is shown. The genomes of *Vibrio cholerae*, *Bacillus anthracis*, *Enterococcus faecalis*, *Streptococcus pneumoniae*, and *Clostridioides difficile* contain *pst* genes in more than one location. The *pst* genes were identified in three to five strains of each species shown, including the strains listed above; in all strains of each species evaluated, the genomic organization of the *pst* genes was consistent with what is shown. Genome accession numbers and locus tags for each strain shown are listed in Table S1.

The *pst-phoU* operon is well conserved throughout the bacterial kingdom, but the genes that comprise the operon vary among species (8). In its most basic form, the operon contains *pstS*, *pstC*, *pstA, pstB*, and *phoU. pstS* encodes a Pi-binding protein that delivers Pi to the importer, where it is actively transported across the membrane and into the cytoplasm (9, 10). *pstC* and *pstA* encode hydrophobic membrane-spanning components of the Pst system that heterodimerize, forming a channel for Pi to enter the cell (11–13). The gene product of *pstB* is identified as an ATP-binding cassette (ABC), which binds and hydrolyzes ATP in order to power the active transport of Pi across the membrane (12, 14). PhoU connects the PstSCAB Pi importer with the phosphate-responsive PhoB/PhoR two-component system and serves as a negative regulator of phosphate signaling, likely through its known interactions with PstB and PhoR (15–17). Interestingly, some species have partial or complete additional copies of the operon located elsewhere in their genomes (8). In addition, a number of gram-positive bacteria have *pst-phoU* operons with two adjacent *pstB* genes (Fig. 1) (8). The roles of these duplicate *pstB* genes, and whether or not they perform identical or dissimilar functions, are not understood (8).

*Enterococcus faecalis* is a gram-positive gastrointestinal commensal and opportunistic pathogen of humans, composing roughly 0.1% of the human gut microbiota (18). Having likely emerged during the Paleozoic Era, during the period that some animal species first left the oceans for land, *E. faecalis* has evolved to exist and thrive in multiple environments (19). Enterococci are highly resistant to desiccation, UV radiation, detergents, disinfectants, bile salts, heat, and high salinity (19–22). Despite the importance of Pi in microbial metabolism and virulence, neither Pi uptake nor the *pst-phoU* locus has been characterized to date in *E. faecalis*. Furthermore, the *E. faecalis pst-phoU* locus has adjacent genes annotated as *pstB1* and *pstB2* (Fig. 1). In this work, we sought to compare and contrast the functionality of *pstB1* and *pstB2* to better understand the role of each gene in *E. faecalis* strain OG1RF with respect to growth, *pst-phoU* locus expression, and Pi uptake. Our results demonstrate that deleting either *pstB* gene from *E. faecalis* results in vastly different phenotypic outcomes, suggesting that these two genes have non-overlapping functions.

## MATERIALS AND METHODS

### Bacterial strains, growth conditions, and reagents

*E. faecalis* strains used in this study are listed in Table S2. Plasmids are listed in Table S3. The *E. faecalis* strain used in this study, OG1RF, contains no mobile genetic elements, so discoveries made in this strain can likely be extrapolated to other *E. faecalis* strains (23). *E. faecalis* strains were cultured in brain heart infusion (BHI) broth (Becton-Dickinson and Co., Franklin Lakes, NJ), on BHI containing 1.5% agar, or in chemically defined medium (CDM) (24, 25). Ten percent and 25% BHI broths were prepared by 1:10 and 1:4 dilutions (vol/vol) of sterile BHI broth, respectively, in sterile Milli-Q-filtered water. Overnight cultures of strains were incubated at 25°C or 37°C under static conditions in ambient air for 16–20 hours, unless otherwise stated. *E. coli* strain DH5α, which was used to propagate plasmids, was grown in Luria Broth (LB), containing 10 g/L tryptone, 5 g/L yeast extract, and 10 g/L sodium chloride (ThermoFisher Scientific, Waltham, MA). LB or Super Optimal broth with Catabolite repression (SOC) was used as recovery medium for *E. coli* following chemical transformation.

Plasmid-containing strains were grown in broth culture or on agar plates containing antibiotics for selection at the following concentrations, unless otherwise specified: 20 µg/mL chloramphenicol, 50 µg/mL carbenicillin, or 100 µg/mL erythromycin. All antibiotics were purchased from Sigma-Aldrich (St. Louis, MO). Restriction enzymes and other enzymes used for recombinant DNA methods were purchased from New England Biolabs (Ipswich, MA). PfuUltra II Fusion DNA polymerase (Agilent, Santa Clara, CA) or high-fidelity Phusion Hot Start II DNA Polymerase (ThermoFisher Scientific) were used for all PCR amplifications performed for strain and plasmid construction. DL-2-amino-3-phosphonopropionic acid (APP), 2-aminoethylphosphonic acid (AEP), (±)-2-amino-4-phosphonobutyric acid (APB), methylphosphonic acid (MPP), and polyphosphate were purchased from Sigma-Aldrich.

### Strain construction

Oligonucleotides are listed in Table S4. Genomic DNA was extracted from *E. faecalis* OG1RF with the DNeasy Blood and Tissue Kit (Qiagen, Inc., Germantown, MD), according to the manufacturer’s instructions. In-frame markerless deletion strains were constructed using previously described allelic exchange methods (26). The Δ*pstB1* deletion construct used for allelic exchange was generated with overlap extension PCR by first amplifying two ~1 kb fragments from OG1RF genomic DNA with primer pairs EF1755(pstB1)-2stepF/EF1755(pstB1)-downR and EF1755(pstB1)-upF/EF1755(pstB1)-2stepR. The two products were annealed together, and second-step amplification was performed with primers EF1755(pstB1)-2stepF/EF1755(pstB1)-2stepR. The resulting ~2 kb product was gel purified, A-tailed with Taq DNA polymerase in ThermoPol buffer (New England Biolabs), and ligated into pGEM T-EZ (Promega, Madison, WI) to generate pGEM-*pstB1*. pGEM-*pstB1* was amplified in *E. coli* DH5α, and the *pstB1* deletion construct was verified with Sanger sequencing. The 2 kb deletion construct was released from the pGEM T-EZ backbone with EcoRI restriction digest, gel purified, and ligated with T4 DNA ligase into pCJK47 that had been digested with EcoRI and dephosphorylated with calf intestinal phosphatase (26). The ligated plasmid was propagated in *E. coli* EC1000 grown on BHI agar or in BHI broth with erythromycin.

The Δ*pstB2* and Δ*phoZ* deletion constructs used for allelic exchange were generated with overlap extension PCR by first amplifying two ~1 kb fragments from OG1RF genomic DNA with primer pairs *pstB2*-upF_BamHI/*pstB2*-downR_SphI and *pstB2*-2stepF/*pstB2*-2stepR (for deletion of *pstB2*) and *phoZ*-upF_BamHI/*phoZ*-downR_SphI and *phoZ*-2stepF/*phoZ*-2stepR (for deletion of *phoZ*). The two products were then annealed together, and a second round of amplification was undertaken using primer pairs *pstB2*-upF_BamHI/*pstB2*-downR_SphI for the Δ*pstB2* construct and *phoZ*-upF_BamHI/*phoZ*-downR_SphI for the Δ*phoZ* construct. These products were digested with restriction enzymes BamHI and SphI. The digested products were ligated with T4 DNA ligase into pCJK218 digested with the same enzymes and propagated in *E. coli* strain DH5α grown on chloramphenicol (27). The *pstB2* and *phoZ* deletion constructs were verified with Sanger sequencing.

For complementation plasmid generation, *pstB1*, *pstB2*, and *phoZ* were amplified from OG1RF genomic DNA using primer pairs *pstB1* Forward Cloning/*pstB1* Reverse Cloning, *pstB2* Forward Cloning/*pstB2* Reverse Cloning, and *phoZ* Forward Cloning/*phoZ* Reverse Cloning, respectively. The pPLK2 plasmid, which contains the strong constitutive p23 promoter from *Lactococcus lactis* and confers chloramphenicol resistance, was used for complementation (28). pPLK2 and the *pstB1* and *pstB2* PCR products were digested with XbaI and HindIII-HF, gel purified, and ligated with T4 DNA ligase. The *phoZ* PCR product was A-tailed with Taq DNA polymerase in ThermoPol buffer and ligated into pGEM T-EZ, which was then digested with SacI, gel purified, and ligated with T4 DNA ligase into similarly digested and purified pPLK2. The resulting plasmids were propagated in *E. coli* DH5α grown in LB broth or on LB agar supplemented with chloramphenicol. Constructs were verified with Sanger sequencing.

### *E. faecalis* electroporation

Cells were grown overnight in Todd-Hewitt broth (THB) (Fisher Scientific, Waltham, MA) at 37°C. The following day, cultures were diluted 1:10 or 1:20 in fresh THB and incubated at 37°C until an OD_600 nm_ of 0.5–1.0 was reached. Cultures were chilled on ice for 15–20 minutes, then pelleted at 3,452 × *g* at 4°C for 15–30 minutes in a Sorvall Legend RT centrifuge (ThermoFisher Scientific). Cells were resuspended in 500 µL lysozyme solution (10 mM Tris pH 8.0, 20% sucrose, 10 mM EDTA, 50 mM NaCl, and 25 µg lysozyme [Sigma-Aldrich]) and incubated at 37°C for 20 minutes. Cells were washed three times in 1 mL ice-cold electroporation buffer (0.5 M sucrose, 10% glycerol), then resuspended in the same buffer and stored at −80°C. Cells were electroporated with purified plasmid DNA using a Gene Pulser Xcell with PC module (Bio-Rad, Hercules, CA), set to 1.6 kV, 200 Ω, 25 µF. Following electroporation, cells were resuspended in 200 µL THB supplemented with 17.1% sucrose and incubated statically at 37°C for 2 hours. Following incubation, cells were spread on BHI agar with appropriate antibiotic selection. Agar plates with bacteria transformed with pCJK218 derivatives were incubated at 28°C–30°C; agar plates for all other plasmid transformations were incubated at 37°C.

### Growth of *E. faecalis* cultures for RNA extraction

Overnight cultures of strains grown in BHI broth with antibiotic selection were diluted to an OD_600 nm_ of 0.01 in 10% BHI. Three milliliters of diluted cells were pipetted into each well of a six-well plate (Corning Inc., Corning, NY) and incubated statically at 37°C for 6 hours. Following incubation, supernatants from each well were removed and pooled together. An aliquot of the pooled supernatants was serially diluted and plated to enumerate colony forming units (CFU) per milliliter. The remaining pooled supernatants were centrifuged at 3,452 × *g* for 20–30 minutes in a Sorvall Legend RT centrifuge. Cell pellets were resuspended in 600 µL of 1× Tris-HCl, pH 8.0. Cells were treated with RNAprotect Bacteria Reagent (Qiagen) per the manufacturer’s instructions. Cell pellets were stored at −80°C until RNA extraction.

### RNA extraction, DNase treatment, cDNA generation, and quantitative PCR

Cell pellets were resuspended in 200 µL of planktonic lysis solution (30 mg/mL lysozyme, 500 units/mL mutanolysin, 10 mM Tris-HCl pH 8.0, and 1 mM EDTA pH 8.0) and incubated at 37°C for 10 minutes. Following incubation, RNA was extracted with the RNeasy Mini Kit (Qiagen) per the manufacturer’s instructions; buffer RLT was prepared with β-mercaptoethanol. Purified RNA was DNase treated using a Turbo-DNA Free kit (ThermoFisher Scientific), and cDNA was generated with the SuperScript III First-Strand Synthesis System for RT-PCR (ThermoFisher Scientific). qPCR was performed using a BioRad CFX96 C1000 Touch Thermal Cycler with SsoAdvanced Universal SYBR Green Supermix (Bio-Rad). Each reaction was performed in technical triplicate, and threshold cycle (Ct) values were averaged for a single biological replicate. Relative gene expression with respect to the reference gene *gyrB* was calculated using the 2^ΔCt^ equation. A Ct value of 30 was set as the limit of detection for all target genes. The greatest Ct value obtained for *gyrB*, the reference gene, was 25. For analysis purposes, all target gene reactions with Ct values greater than 30 were set to 30.

### Amplification of intergenic regions in the *pst-phoU* locus

PCR primers (Table S4) were designed to generate amplicons of ~100 bp–300 bp that span the intergenic regions between the genes predicted to comprise the *E. faecalis* OG1RF *pst-phoU* locus, as well as the upstream and downstream flanking genes. RNA was isolated from mid-log phase planktonic OG1RF cells grown in BHI. RNA was DNase treated and used for cDNA generation, as described above. The resulting cDNAs were used as templates in PCR reactions with *Taq* DNA Polymerase in ThermoPol buffer (New England Biolabs). Amplicons were separated on a 1.5% Tris-Acetate-EDTA (TAE) agarose gel stained with ethidium bromide and visualized with UV light.

### Growth curves

Overnight cultures grown in BHI with appropriate antibiotics, as needed to select for plasmids, were washed in 1× Tris-HCl, pH 8, and resuspended in either CDM or BHI to an OD_600 nm_ of 0.1 or 0.01 for strains grown at 21°C–25°C or 37°C, respectively. Two hundred microliters of either samples or blanks, with appropriate selective antibiotics added, was pipetted into tissue culture-treated, flat-bottomed 96-well microtiter plates with six wells used per strain sample (Corning). Outer wells of the microtiter plates were filled with 200 µL/well of sterile water in order to maintain humidity through the course of incubation. Plates were placed in a BioTek Synergy HTX Multi-Mode Reader (Agilent) incubated at either 21°C–25°C (room temperature) or 37°C for 16 hours, with OD_600 nm_ measurements taken every 30 minutes immediately following a 5 second shake cycle. The technical replicates of the same strains were averaged together, from which the average of the blank control technical replicates was subtracted to generate a single biological replicate of the growth curve. Three biological replicates were performed on separate days.

### Pi uptake assays

Pi uptake from supernatants was measured using a modification of a previously described method (29). Bacteria from colonies grown overnight at 37°C were incubated statically either for 4 hours or overnight in 25% BHI with appropriate selective antibiotics at 37°C. Cells were washed twice with Pi-free CDM that contained no glucose, then resuspended to an OD_600 nm_ of 0.5 in Pi-free CDM containing 0.01 g/mL glucose. Cells were incubated for 2 hours at 37°C. Following incubation, 750 µL of cells were mixed with 750 µL of 10.5 µM K_2_HPO_4_ and incubated for 1, 5, 10, 30, or 60 minutes at room temperature. One hundred microliters was removed from each sample/time point, serially diluted, and plated to enumerate CFU per milliliter. A positive control of 750 µL of Pi-free CDM with no bacteria mixed with 750 µL of 10.5 µM K_2_HPO_4_ (diluted in Pi-free sterile water) and a blank of 750 µL of Pi-free CDM with no bacteria mixed with 750 µL of Pi-free sterile water were used. Positive and negative control samples were incubated for the same amount of time as the paired strain samples. Following incubation, 1 mL of cell/K_2_HPO_4_ mix was sterilized by passing through a Whatman Uniflo 13 mm 0.2 µmpolyethersulfone filter (Cytiva Life Sciences, Emeryville, CA), and the filtrate was saved. Three technical replicates of 100 µL/well of the filtered medium taken from each strain at each time point were pipetted into the wells of a flat-bottomed 96-well Corning microtiter plate. To determine the remaining Pi concentration in the filtrate, the Malachite Green Phosphate Assay Kit (Sigma-Aldrich) was used in accordance with the manufacturer’s instructions. A phosphate concentration ladder provided by the manufacturer and filtered positive and negative controls were also measured. Following incubation with the kit reagents, the OD_620 nm_ for all samples, controls, and ladder controls was collected using a BioTek Synergy HTX Multi-Mode Reader. The average optical density of the technical replicate values was calculated, then the averaged blank OD_620 nm_ was subtracted. This value was converted into a concentration through the use of the kit-provided phosphate concentration ladder and used to calculate the exogenous Pi in the medium. The medium exogenous Pi concentration was subtracted from the known starting Pi, which was based on the calculated concentration of the positive control. Three biological replicates for all strains and time points were performed.

### Colorimetric detection of alkaline phosphatase (AP) activity

A modification of a qualitative AP activity assay described previously was used (30). Briefly, strains were streaked on BHI agar containing chloramphenicol and 100 µg/mL of the micronized p-toluidine salt form of 5-bromo-4-chloro-3-indoxyl phosphate (XP) (GoldBio, St. Louis, MO). Plates were incubated overnight at 37°C and then imaged with a Pixel 6 camera (Google, Mountain View, CA) with illumination from a Neewer RL-12 LED Ring Light (Shenzhen Neewer Technology, Guangdong, China).

### Alkaline phosphatase activity assays

Overnight cultures of strains grown in BHI with appropriate antibiotic selection were diluted to an OD_600 nm_ of 0.2 in BHI, then incubated at 37°C for 90 minutes to reach OD_600 nm_ ~0.5. Supernatants were collected and saved, and cells were washed once in 1 M Tris-HCl pH 8.0, then resuspended in 1 mL of 1 M Tris-HCl pH 8.0. A 100 µL aliquot of the suspension was serially diluted and plated for CFU per milliliter enumeration. One hundred microliter aliquots of the cell suspension and the supernatants were diluted in 800 µL AP buffer (1 M Tris-HCl pH 8.0, 0.1 mM ZnCl_2_), mixed with 100 µL of 0.4% p-nitrophenyl phosphate (pNPP) (VWR, Radnor, PA), and incubated at 37°C for 10 minutes. One hundred twenty microliters of a 1:5 mix of 0.5 M EDTA to 1 M KH_2_PO_4_ was used to stop the reaction after 10 minutes (31). Two technical replicates, each containing 100 µL of reaction mixture from each strain, were pipetted into the wells of a tissue culture-treated, flat-bottomed 96-well microtiter plate (Corning). The OD_405 nm_ and OD_600 nm_ were measured on a BioTek Synergy HTX Multi-Mode Reader for each sample. The technical replicate values were averaged, and AP activity was calculated using the following equation modified from Zhang et al.: $AP\;activity=1,000*\frac{OD_{405}}{\left(OD_{600}\;\times \;volume\;\times \;reaction\;time\right)}$ (32). Three biological replicates were performed.

### Evaluation of phosphorus sources to support growth of OG1RF, *pstB1*, and *pstB2* deletion mutants

To evaluate phosphorus-containing compounds capable of supporting the growth of *E. faecalis pstB1* and *pstB2* mutants in CDM, Phenotype MicroArray PM4A plates for phosphorus and sulfur utilization assays were procured from Biolog (Biolog Inc., Haywood, CA). Bacterial strains were grown statically in BHI broth for 16 hours, washed in 1× Tris-HCl, pH 8.0, then resuspended to an OD_600 nm_ of 0.01 in CDM containing no inorganic phosphorus. The PM4A plates were inoculated with 100 µL per well of the resuspended cultures, then incubated for 16 hours at 37°C in a sealed plastic food storage container lined with damp paper towels in order to maintain humidity. The OD_600 nm_ of each well was measured on a BioTek Synergy HTX Multi-Mode Reader after 16 hours growth. Four to five biological replicates were performed for each strain, with outlier replicates removed after application of the Grubbs Test online calculator (GraphPad Software, Boston, MA).

Growth curves with other phosphorus sources were carried out in CDM as described above. Phosphonate compounds were added to CDM lacking other phosphorus sources at a final concentration of 0.2 mM. Polyphosphate was added to CDM lacking other phosphorus sources at a final concentration of 0.7% (wt/vol).

## RESULTS

### Confirmation that the *E. faecalis* OG1RF *pst-phoU* locus is expressed as an operon

**Fig 2:**
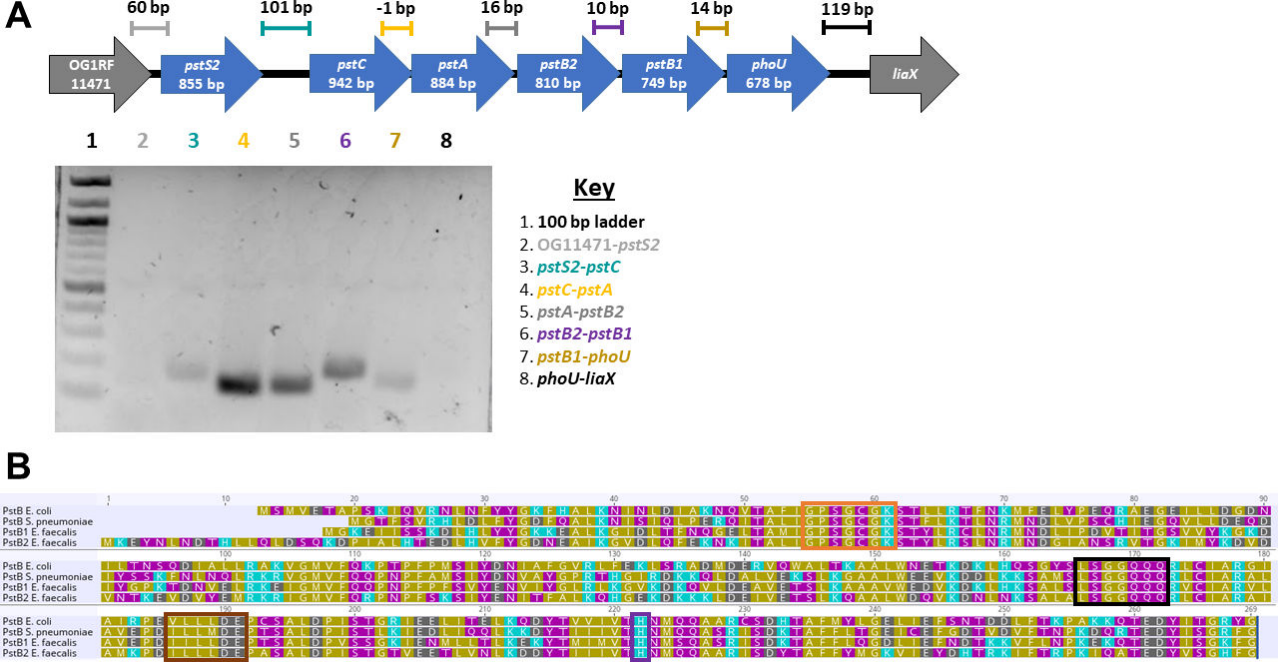
The *E. faecalis pst-phoU* locus is transcribed as an operon and encodes PstB1 and PstB2, which are likely ATPases. (A) Primers were designed to amplify the intergenic regions of the *E. faecalis* OG1RF *pst-phoU* locus (*pstS2* to *phoU*), as well as the upstream and downstream flanking genes (OG1RF_11471 and *liaX*, respectively). Amplification of cDNA from the intergenic regions located between *pstS2* and *phoU* was observed. There was no amplification of cDNA from the intergenic regions between OG1RF_11471 and *pstS2* or *phoU* and *liaX*. The diagram above the gel image shows the organization of the *pst-phoU* region in *E. faecalis* OG1RF. The sizes of the intergenic regions and the approximate regions amplified are indicated. Gel image is representative of four biological replicates. (B) Alignment of amino acid sequences for PstB from *E. coli* BW25113 strain K-12 and *Streptococcus pneumoniae* strain R6, which are known ATPases, and PstB1 and PstB2 from *E. faecalis* strain OG1RF. Sequence motifs characteristic of ATPases are identified by colored boxes as follows: orange, Walker A motif; brown, Walker B motif; black, LSGGQ motif; and purple, H-loop. Individual amino acids are colored as follows: goldenrod, hydrophobic side chain; teal, positively charged side chain; gray, negatively charged side chain; and purple, polar uncharged side chain. The alignment was generated with Geneious Prime software (version 2023.0.4).

The *pst-phoU* locus in *E. faecalis* has not been definitively shown to be organized as an operon as it is in other bacteria, including *E. coli,* multiple streptococcal and *Bacillus* species, and *Nostoc punctiforme* (8, 33–35). In order to investigate this, primer pairs were designed to amplify the intergenic regions spanning from *pstS2* through *phoU*, as well as the adjacent intergenic regions flanking the *pst-phoU* locus (OG1RF_11471-*pstS2* and *phoU-liaX*; Fig. 2A). cDNA was used as a template, with the rationale that if the *pst-phoU* locus in *E. faecalis* is expressed as a polycistronic RNA, then all genes and intergenic regions should be co-transcribed. Figure 2A shows amplification of the intergenic regions from *pstS2* through *phoU*, indicating the presence of reverse-transcribed polycistronic mRNA and supporting the hypothesis that the *pst-phoU* locus in *E. faecalis* is organized as an operon. There was no amplification of either the OG1RF_11471-*pstS2* or the *phoU-liaX* intergenic regions, suggesting that these flanking genes are not included in the *pst-phoU* polycistronic transcript.

### The *E. faecalis pst-phoU* locus contains adjacent genes annotated to encode ATP-binding proteins

The *E. faecalis* OG1RF *pst-phoU* operon contains adjacent genes named *pstB1* and *pstB2* (Fig. 1), which encode protein products that are 60% identical/79% similar at the amino acid level based on Basic Local Alignment Search Tool (BLAST) analysis (36). PstB has been annotated as an ATPase in a number of species. ATPases contain two ATP-binding domains with characteristic motifs, called the Walker A and Walker B motifs. The Walker A motif is composed of the sequence GXXGXGK(S/T), where X can be any amino acid (37–39). The Walker B motif is composed of the sequence hhhhDE, where h denotes any hydrophobic amino acid (38, 39). ATPases also contain an ABC transporter-specific signature sequence of LSGGQ and a conserved histidine residue (H-loop) (35, 38–40). To assess whether *E. faecalis* OG1RF PstB1 and PstB2 (NCBI nucleotide database accession number: CP025020) both have sequence characteristics that are identifiable in known ATPases, we compared the two *E. faecalis* protein sequences with PstB ATPases from *E. coli* BW25113 strain K-12 (accession number: NZ_CP009273) and *Streptococcus pneumoniae* strain R6 (accession number: AE007317.1). The PstB protein of *E. coli* BW25113 strain K-12 is 51% identical/72% similar to PstB1 and 50% identical/72% similar to PstB2. *S. pneumoniae* has two *pst-phoU* loci, one of which contains a single copy of *pstB* (Fig. 1) that is 62% identical/78% similar to PstB1 and 58% identical/80% similar to PstB2. We were able to identify the four ATPase-specific sequence motifs in both *E. faecalis* PstB1 and PstB2 (Fig. 2B). The Walker A, LSGGQ, and H-loop motifs were identical among the four aligned sequences, while the hydrophobic amino acids in the N-terminal region of the Walker B motif sequence varied among the four proteins. These results suggest that PstB1 and PstB2 are ATPase components of the *E. faecalis* Pst ABC transporter.

### *pstB1* and *pstB2* deletion mutants exhibit growth defects when grown in CDM but not BHI

**Fig 3:**
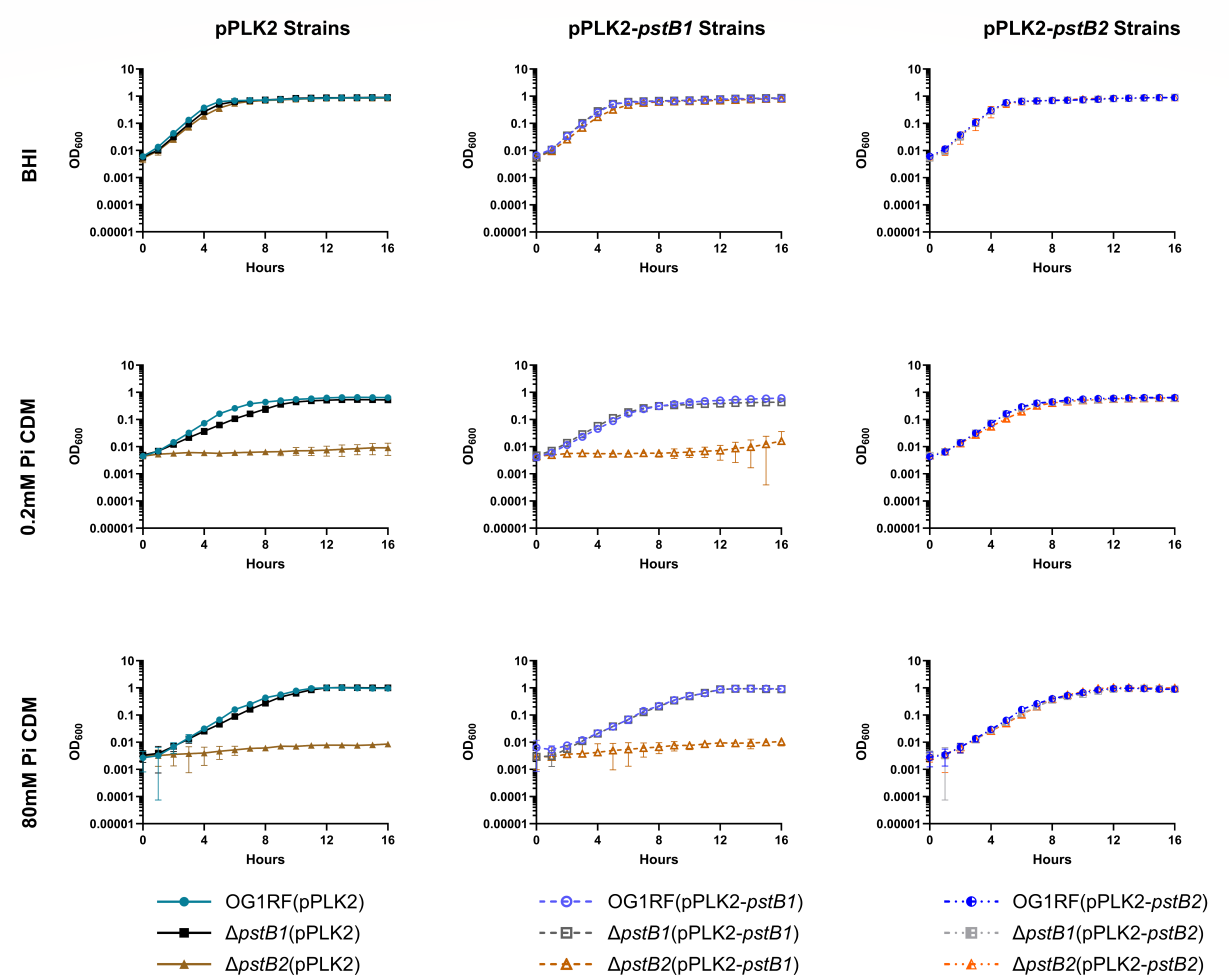
Δ*pstB1* exhibits a growth defect in low-Pi CDM, and CDM does not support growth of Δ*pstB2* at 37°C. Bacterial growth was monitored over time for strains grown in BHI (top row), CDM containing 0.2 mM Pi (low-Pi CDM; middle row), and CDM containing 80 mM Pi (high-Pi CDM; bottom row). Graphs in the first column show only strains that carry the empty vector (pPLK2). The second and third columns show strains that express *pstB1* or *pstB2*, respectively, *in trans* from pPLK2. Each data point is the mean of three biological replicates. Error bars show the standard deviation. Statistical analyses for the middle and bottom rows are found in Tables S5 and S6, respectively.

We next generated in-frame, markerless single-gene deletion strains of *pstB1* and *pstB2* in the *E. faecalis* OG1RF genetic background. Wild-type copies of *pstB1* and *pstB2* were each expressed *in trans* in the wild-type and mutant strains from under the control of the strong constitutive p23 promoter (28). All strains grew well when cultured in BHI broth, an undefined nutrient-rich medium, and incubated at either 37°C or 25°C (Fig. 3A; Fig. S1, respectively).

The Δ*pstB1*(pPLK2) strain exhibited a lag in growth compared to the wild-type strain in low-Pi (0.2 mM) CDM at 37°C and 25°C (Fig. 3B; Fig. S1 middle row, respectively). The growth lag was reduced, but still present, in high-Pi (80 mM) CDM at both temperatures (Fig. 3;Fig. S1, bottom row). Expression of a wild-type copy of *pstB1 in trans* in the Δ*pstB1* strain largely restored the growth kinetics in both low- and high-Pi CDM (Fig. 3; Fig. S1, middle and bottom rows). Interestingly, providing a wild-type copy of *pstB2 in trans* in the Δ*pstB1* strain, generating a *pstB2* merodiploid strain, restored wild-type growth kinetics in both low and high Pi-containing CDM concentrations at 37°C and 25°C (Fig. 3; Fig. S1, middle and bottom rows). Unexpectedly, the Δ*pstB2*(pPLK2) strain was unable to grow in CDM regardless of Pi concentration or temperature (Fig. 3; Fig. S1, middle and bottom rows). Only expression of *pstB2 in trans* in the Δ*pstB2* genetic background was capable of rescuing growth of the strain in CDM; expression of *pstB1 in trans* in the Δ*pstB2* background was unable to rescue the growth phenotype of the mutant to wild-type (Fig. 3; Fig. S1, middle and bottom rows).

### Deletion of either *pstB1* or *pstB2* significantly increases transcription of the remaining *pstB* gene

**Fig 4:**
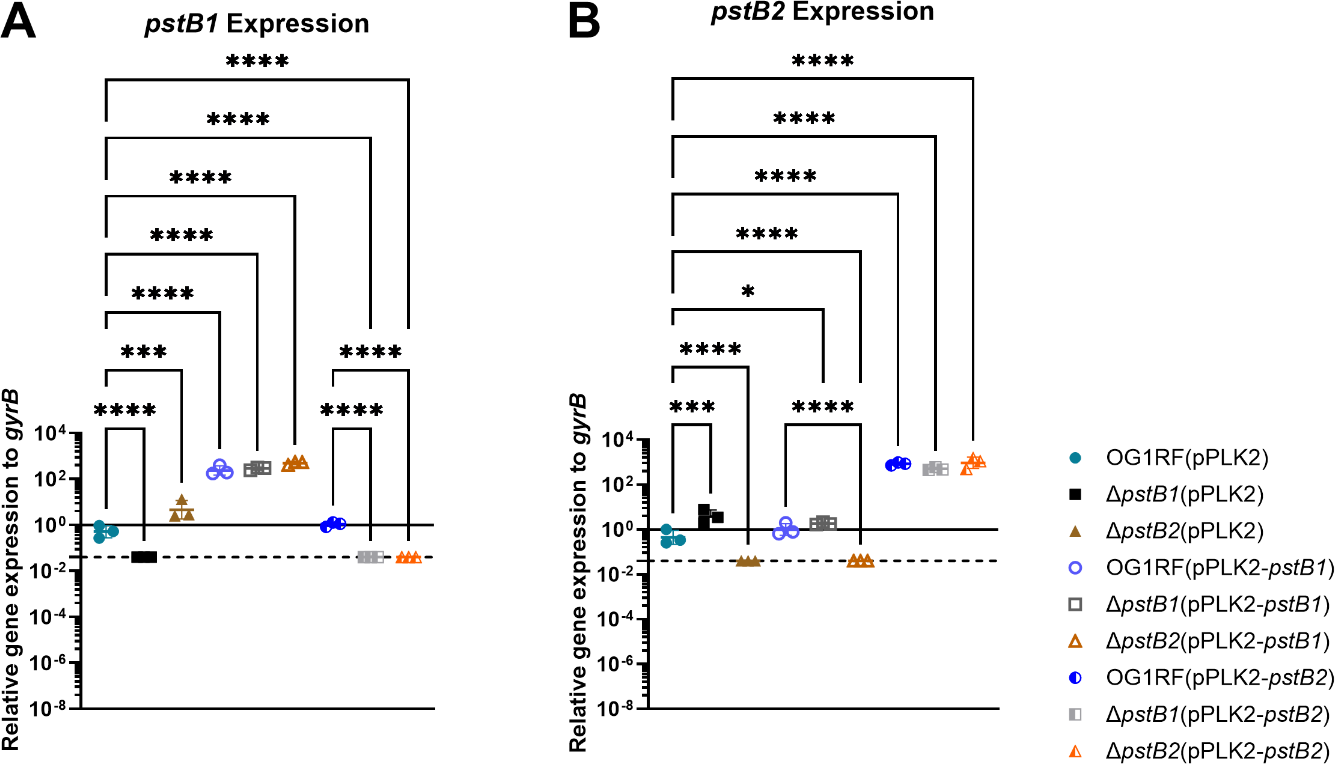
Deletion of either *pstB1* or *pstB2* significantly increases expression of the other *pstB* gene, and overexpression of *pstB2* in the Δ*pstB*2 strain is associated with decreased expression of *pstB1*. RNA isolated from bacterial strains incubated in 10% BHI for 6 hours was reverse transcribed and analyzed via qPCR for expression of (A) *pstB1* or (B) *pstB2* relative to the reference gene *gyrB*. The solid black line indicates a relative gene expression level of 1. The dashed black line indicates the lowest possible value for relative gene expression based on the limit of detection of the target genes (see Materials and Methods). Each data point represents an independent biological replicate. Horizontal bars show the mean; error bars indicate the standard deviation. One-way analysis of variance with Tukey’s correction: *, *P* < 0.05; ***, *P* < 0.001; ****, *P* < 0.0001.

The cyanobacterium *Nostoc punctiforme* has four copies of *pstB* across three distinct loci. Deletion of the *N. punctiforme pstB1* allele was shown to affect expression of two of the other three *pstB* genes that are located across its genome (34). Although the *E. faecalis pstB1* and *pstB2* alleles are in the same locus and are expected to be co-transcribed based on the data in Fig. 2A, there is evidence of alternative internal promoters in the *E. coli pst* operon (41). Therefore, we evaluated if deletion of *pstB1* or *pstB2* would affect transcription of the other *pstB* gene. Since the Δ*pstB*2 strain did not replicate in CDM (Fig. 3; Fig. S1), we extracted RNA from cells that were incubated in 10% BHI for 6 hours. Ten percent BHI has less Pi than undiluted BHI and supported a minimal number of replications of all strains (Fig. S2A), thereby creating a condition that would likely push the cells toward a phosphorus-starved state while maintaining enough nutrients to support the predicted increased expression of Pi acquisition machinery (e.g., the *pst-phoU* operon). Due to the low culture density resulting from the 10% BHI (Fig. S2, top row), cultures were collected after 6 hours of incubation to maximize the number of cells harvested. In the *pstB2* deletion mutant, *pstB1* expression was significantly increased compared to the wild-type (Fig. 4A). The converse, increased expression of *pstB2* in the Δ*pstB1* deletion strain, was likewise observed (Fig. 4B). The data also confirmed that strains containing pPLK2-*pstB1* or pPLK2-*pstB2* had significantly increased RNA levels of the corresponding gene (Fig. 4A and B). Finally, overexpression of *pstB2* was associated with decreased *pstB1* expression in the Δ*pstB*2(pPLK2-*pstB2*) strain relative to OG1RF(pPLK2), but the same was not observed with the OG1RF(pPLK2-*pstB2*) strain (Fig. 4A). Conversely, overexpression of *pstB1* had no effect on expression of *pstB2* in any of the strains (Fig. 4B). The reason why *pstB1* expression is significantly decreased in the Δ*pstB*2 complementation strain but not the *pstB2* merodiploid strain [OG1RF(pPLK2-*pstB2*)] is not fully clear. Overall, the data in Fig. 4 suggest that there may be a difference in the regulation of *pstB1* and *pstB2*.

### Pi uptake is differentially impaired in the *pstB* deletion mutants

**Fig 5:**
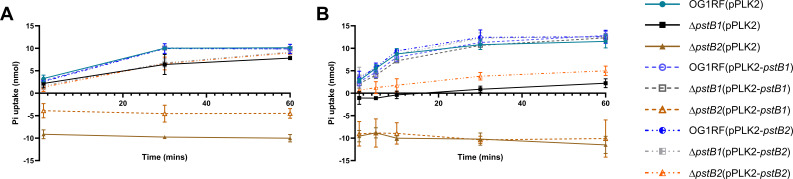
Δ*pstB1* exhibits diminished Pi uptake compared to wild-type, while medium in Δ*pstB2*-containing samples rapidly accrued more Pi than was initially added. Cultures of each strain were grown in 25% BHI for (A) 4 hours or (B) overnight, then incubated in Pi-free medium for 2 hours. Following this Pi starvation step, all strains were mixed with K_2_HPO_4_ and incubated for 1, 5, 10, 30, or 60 minutes. Cells were removed by filtration, then exogenous Pi remaining in the medium was measured. Pi uptake was calculated as described in the Materials and Methods. Data and error bars at each time point show the mean and standard deviation, respectively, of three biological replicates. Figure S3D shows the same data with the strains separated by empty vector, pPLK-*pstB1*, and pPLK-*pstB2*.

We next compared the roles of *pstB1* and *pstB2* in *E. faecalis* Pi uptake with a malachite green-based assay that specifically detects Pi. Strains were grown for either 4 hours (Fig. 5A) or 24 hours (Fig. 5B) in 25% BHI, which was chosen because it supports some replication of the *E. faecalis* strains (Fig. S2, bottom row) while also providing less phosphorus compared to undiluted BHI. Following growth in 25% BHI, the bacterial strains were washed and further starved of phosphorus for 2 hours by incubating in Pi-free CDM. The bacterial cells were then incubated in K_2_HPO_4_, and Pi uptake was measured over time (Fig. 5). OG1RF(pPLK2), OG1RF(pPLK2-*pstB1*), and OG1RF(pPLK2-*pstB2*) displayed similar Pi uptake kinetics following both phosphorus-starvation time intervals, reaching a maximum of ~10 nmol by 60 minutes. Pi uptake in the Δ*pstB1*(pPLK2) strain was reduced compared to the wild-type strain under both conditions. Moreover, when Δ*pstB1*(pPLK2) was grown overnight in 25% BHI, uptake was close to 0 nmol for the first 30 minutes after Pi was introduced to the bacteria and thereafter increased by only a fraction of the amount that the wild-type strain increased (Fig. 5B). Expression of either *pstB1* or *pstB2 in trans* in the Δ*pstB1* strain restored Pi uptake to wild-type levels. Unexpectedly, the medium containing Δ*pstB2*(pPLK2) and Δ*pstB2*(pPLK2-*pstB1*) contained more Pi than the starting concentration after just 1 minute and stayed level through the duration of the experiment, indicating that neither of these strains took up Pi. The amount of Pi measured in the medium was lower for the Δ*pstB2*(pPLK2-*pstB1*) strain compared to the Δ*pstB2*(pPLK2) strain in the cells grown in 25% BHI for 4 hours (Fig. 5A), suggesting the possibility that *pstB1* expression partially rescued the Pi uptake deficiency. However, when the same strain was grown overnight in 25% BHI (Fig. 5B), there was no difference in the amount of Pi in the medium compared to the Δ*pstB2*(pPLK2) strain. Expression of *pstB2 in trans* in the Δ*pstB2* background resulted in a Pi uptake phenotype similar to the Δ*pstB1* mutant in both conditions. Importantly, each time point of this assay contained an equivalent number of viable cells for each strain (Fig. S3A and B). These results indicate that the excess Pi in the Δ*pstB2*(pPLK2) and Δ*pstB2*(pPLK2-*pstB1*) reactions was not the result of cell lysis.

### AP activity and expression are differentially increased in the Δ*pstB1* and Δ*pstB2* mutant backgrounds

**Fig 6:**
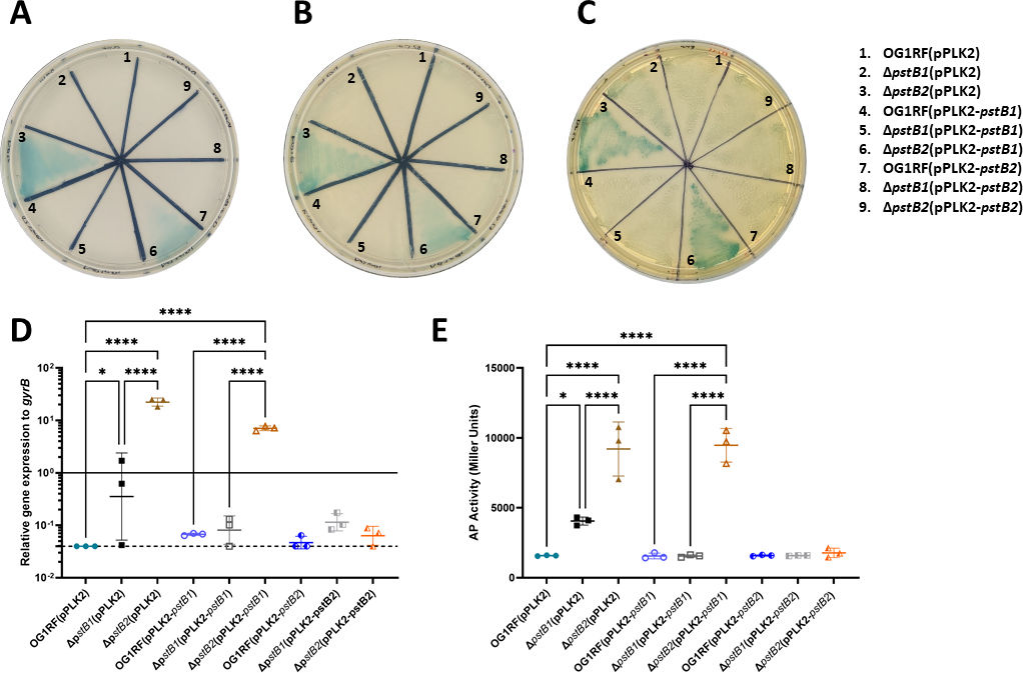
AP activity and *phoZ* expression are highly increased in the Δ*pstB2* strain compared to the wild-type strain. (A–C) Colorimetric-based phenotypes of AP activity on (A) 10%, (B) 25%, or (C) 100% BHI agar containing 100 µg/mL XP. A darker blue color indicates more AP activity. The image shown is representative of three biological replicates. (D) RT-qPCR measurement of relative expression of *E. faecalis phoZ*, which encodes AP, as compared to the reference gene *gyrB*. RNA was collected from strains incubated in 10% BHI for 6 hours. The solid black line indicates a relative gene expression level of 1. The dashed black line indicates the lowest possible value for relative gene expression based on the limit of detection of the target genes (see Materials and Methods). Each symbol represents an independent biological replicate. Horizontal bars show the mean; error bars indicate the standard deviation. (E) Quantitative measurement of AP activity in cell-associated fractions by cleavage of the colorimetric substrate pNPP. Each symbol represents an independent biological replicate. Horizontal lines show the mean; error bars indicate the standard deviation. For (D) and (E), one-way analysis of variance with Tukey’s correction: *, *P* < 0.05; ****, *P* < 0.0001.

In *E. coli,* periods of Pi starvation have been shown to increase synthesis of AP (also called PhoA or Bap) over 1,000-fold (33). Based on the observation that neither the Δ*pstB1* nor the Δ*pstB2* mutants were able to take up Pi as efficiently as the wild type (Fig. 5), we hypothesized that AP may be upregulated in these mutants as a means to overcome a Pi deficit. Qualitative analysis of AP activity on 10%, 25%, and 100% XP-supplemented BHI agar suggested that the Δ*pstB2*(pPLK2) and Δ*pstB2*(pPLK2-*pstB1*) strains displayed the strongest AP activities of all the strains tested (Fig. 6A through C, sectors 3 and 6, respectively). There was no color produced by the wild type, nor the *pstB1* or *pstB2* merodiploid strains in the wild-type background (Fig. 6A through C, sectors 1, 4, and 7, respectively). The Δ*pstB2* mutant phenotype was restored to that of the wild-type strain only when *pstB2* was expressed *in trans* in the strain (Fig. 6A through C, sector 9); *in trans* expression of *pstB1* from the pPLK2 plasmid in the strain did not affect the mutant phenotype (Fig. 6A through C, sector 6). The Δ*pstB1*(pPLK2) strain displayed a slight blue coloration (Fig. 6A through C, sector 2; most visible on 100% BHI agar in panel C), and this phenotype was not present when either *pstB1* or *pstB2* was expressed *in trans* in the Δ*pstB1* genetic background (Fig. 6A through C, sectors 5 and 8).

The product of the *E. faecalis phoZ* gene has been characterized as an AP (31). As follow-up to the qualitative AP activity assay (Fig. 6A through C), we measured expression of *phoZ* (locus OG1RF_12255) in our OG1RF wild-type and mutant strains (Fig. 6D). We observed increased levels of expression of *phoZ* in Δ*pstB1* and Δ*pstB2* compared to wild-type. Of the two knockout mutants, the Δ*pstB2* strain displayed a greater level of *phoZ* expression, which is consistent with the qualitative AP activity results (Fig. 6A through C). Expression of wild-type copies of *pstB1* or *pstB2* from the pPLK2 plasmid in the Δ*pstB1* background restored expression of *phoZ* to wild-type levels. However, similarly to what was observed above (Fig. 3 to 5 and 6A through C), only expression of *pstB2* in the Δ*pstB2* strain restored *phoZ* expression back to wild-type levels; *phoZ* expression in Δ*pstB2*(pPLK2-*pstB1*) was similar to the Δ*pstB2*(pPLK2) strain.

In gram-negative bacteria, AP activity is highest in the periplasm (42–44). For gram-positive bacteria, AP is relegated to the outer surface of the plasma membrane (45, 46). AP can also be secreted from bacterial cells (47). In order to identify the location of enzymatically active PhoZ in *E. faecalis* and quantitate the relative amount of AP produced by each strain, we used a pNPP-based colorimetric assay to measure AP activity in the cell-associated fractions of mid-log phase cells (Fig. 6E) and the associated culture supernatants (Fig. S4). We observed increased AP activity in the Δ*pstB1* and Δ*pstB2* strains compared to wild-type, with Δ*pstB2* exhibiting the highest amount of AP activity. Expression of wild-type copies of either *pstB1* or *pstB2* in Δ*pstB1* restored AP activity to wild-type levels. The Δ*pstB2*(pPLK2-*pstB2*) strain also exhibited AP activity that was similar to that of the wild-type strain, but the Δ*pstB2*(pPLK2-*pstB1*) strain retained a high level of AP activity. The overall amount of AP activity was substantially lower in the culture supernatants (Fig. S4A), with the Δ*pstB2*(pPLK2-*pstB1*) supernatant having the most AP activity. Enumeration of CFU per milliliter from the source cultures used in the pNPP-based assay confirmed that there was no significant difference between the cell counts of the different strains (Fig. S4B). Taken together, these results indicate that the *phoZ*-encoded AP is primarily associated with *E. faecalis* cells rather than being secreted into the culture supernatant at high levels. This finding is consistent with SignalP 6.0 and previous predictions that *E. faecalis phoZ* is a lipoprotein (48, 49).

### AP is partially responsible for the rapid accrual of external Pi observed in the growth medium of Δ*pstB2*-containing cultures

**Fig 7:**
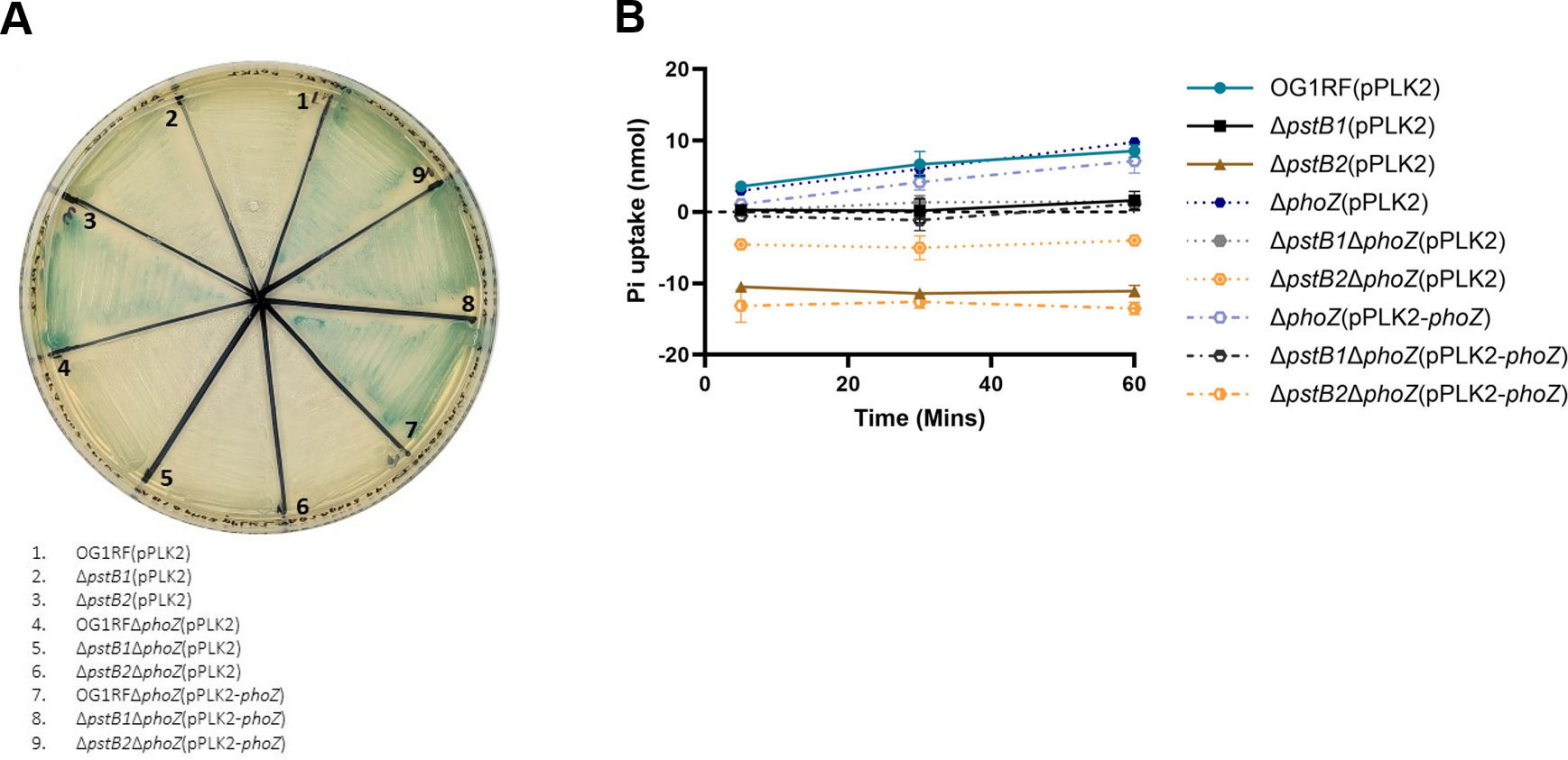
Deletion of *phoZ* reduces the amount of Pi that accrues in the medium of Δ*pstB2*-containing samples. (A) Colorimetric-based phenotypes of AP expression on agar containing 100 µg/mL XP for Δ*phoZ* mutant strains. The image shown is representative of three biological replicates. (B) Overnight cultures of each strain were starved of phosphate for 2 hours, then were mixed with K_2_HPO_4_ and incubated for 5, 30, or 60 minutes. One milliliter was removed from each incubated time point and filter sterilized. Exogenous Pi remaining in the medium was measured, and Pi uptake was calculated as described in the Materials and Methods. Data and error bars at each time point show the mean and standard deviation, respectively, of three biological replicates.

The results shown in Fig. 5 and 6 led us to hypothesize that the increased Pi present in the medium of the Δ*pstB2* strain (Fig. 5) may be due to the strain’s increased AP activity (Fig. 6). Specifically, the increased AP activity could result in enhanced release of Pi from phosphate-containing molecules released from the cells via secretion or upon lysis. In order to test this hypothesis, we generated in-frame deletions of *phoZ* in the wild-type, Δ*pstB1*, and Δ*pstB2* strain backgrounds. Differences in growth between the strains were minimal (Fig. S5). Deletion of *phoZ* in the Δ*pstB2* background rendered the strain unable to break down XP in a qualitative assay of AP activity (Fig. 7A, sector 3 versus sector 6), while expression of *phoZ in trans* led to enhanced AP activity in the wild-type and both Δ*pstB* mutants (Fig. 7, sectors 7–9).

Deletion of *phoZ* from OG1RF and Δ*pstB1* had no effect on the Pi uptake phenotypes (Fig. 7B). Similarly, expression of *phoZ in trans* in the same two strain backgrounds did not alter the Pi uptake phenotypes. In contrast, deletion of *phoZ* in the Δ*pstB2* background reduced the amount of exogenous Pi compared to the Δ*pstB2* parental strain; however, by the 5 minute time point, the medium had still accrued more Pi than was initially added (Fig. 7B). Expression of *phoZ in trans* in the Δ*pstB2*Δ*phoZ* strain restored the phenotype of the parent strain [Δ*pstB2*(pPLK2)]. Similar numbers of viable cells were used for each strain, indicating that the observed results were most likely due to genetic differences between the strains (Fig. S3C). Therefore, from these results, we conclude that the increased activity of the *phoZ*-encoded AP in the Δ*pstB2* strain partially accounts for the accumulation of Pi in the culture medium that we unexpectedly observed in Fig. 5.

### Investigation of alternate sources of phosphorus capable of sustaining growth of Δ*pstB2* in CDM

The observation that the Δ*pstB2* mutant was incapable of growth in CDM regardless of the concentration of Pi present, but was capable of reaching stationary phase in BHI broth (Fig. 3), suggests that BHI contains a phosphorus source that the Δ*pstB2* mutant can use for its metabolic needs in lieu of Pi. The CDM recipe used in this work contains no other sources of phosphorus beyond Pi, except for minute traces of inorganic phosphate in the form of NADP and vitamin B_12_. Concentrations of these two molecules are roughly 0.25 mg and 0.01 mg per 0.01 L of CDM, respectively (24, 25). No growth was observed for any of the strains used in this study when they were incubated in CDM lacking any other source of phosphorus (beyond the trace amounts supplied by the base medium) (data not shown). These results led us to hypothesize that Δ*pstB2* was utilizing an alternate, non-Pi source of phosphorus in order to meet its metabolic requirements when incubated in BHI.

**Fig 8:**
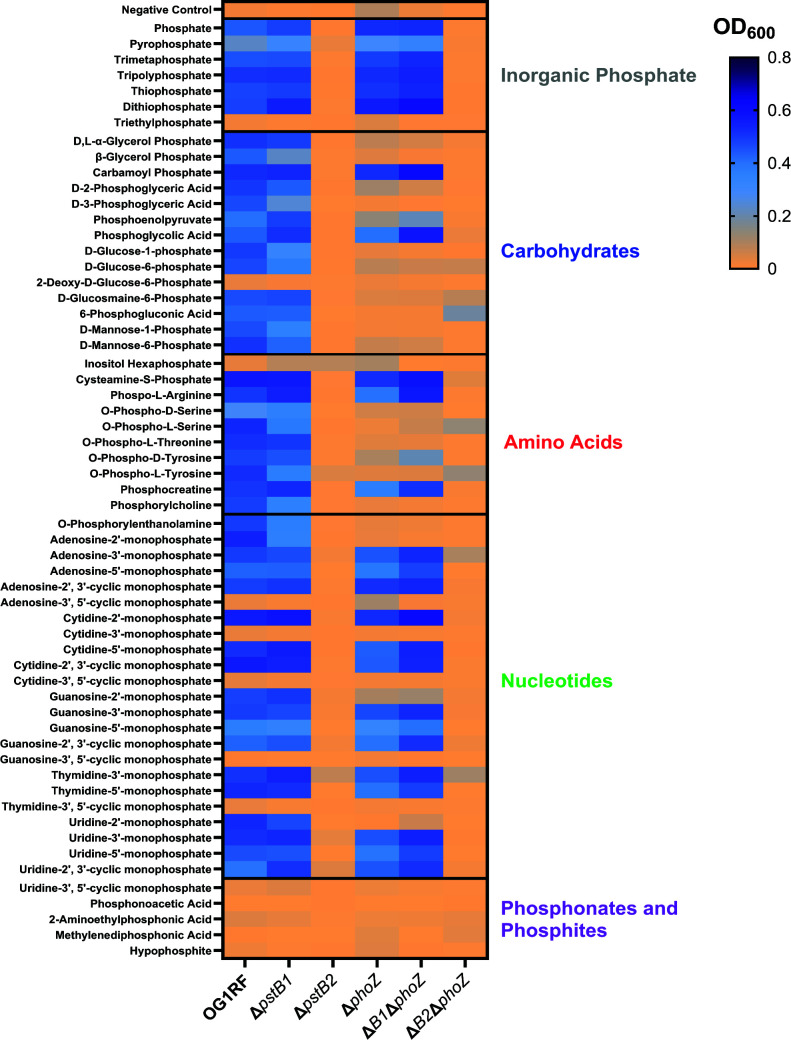
Identification of phosphorus-containing compounds that support growth of the wild-type, Δ*pstB1*, or Δ*pstB2* strains in CDM. Biolog PM4A plates containing 59 different phosphorus-containing molecules were inoculated with either wild-type, Δ*pstB1*, Δ*pstB2*, Δ*phoZ*, Δ*pstB1*Δ*phoZ*, or Δ*pstB2*Δ*phoZ* suspended in CDM with 0.0 mM Pi. Bacterial growth was measured by reading the OD_600 nm_ of each well after incubation in a humidity chamber at 37°C for 16 hours. The 59 separate compounds are grouped as inorganic phosphorus molecules (black) and organic phosphorus molecules that are further subdivided into carbohydrates (blue), amino acids (red), nucleotides (green), and phosphonates and phosphites (purple). *N* = 3–4 biological replicates for OG1RF, Δ*pstB2,* and the three *phoZ* deletion strains, and 4–5 biological replicates for Δ*pstB1*. Outliers identified by the application of the Grubb’s test were excluded from the data set.

We screened a library of 59 phosphorus-containing molecules present on Biolog PM4A plates in order to identify which phosphorus-containing compounds could support growth of OG1RF, Δ*pstB1*, and Δ*pstB2* in CDM lacking exogenous phosphorus. The library contained both inorganic phosphorus, including Pi, and organic phosphorus compounds that were composed of carbohydrates, amino acids, nucleotides, phosphonates, and hypophosphite (Fig. 8). The wild-type and Δ*pstB1* strains had similar patterns of growth when incubated in CDM with the phosphorus-containing compounds. Meanwhile, the maximum end point OD_600_ observed for Δ*pstB2* for any of the compounds was ~0.2, and this only occurred in the wells containing inositol hexaphosphate, thymidine 3´-monophosphate, and uridine-2´,3´-cyclic monophosphate. However, in follow-up experiments, inositol hexaphosphate was insufficient at supporting growth of the Δ*pstB2* strain when it was used as the sole phosphorus source in CDM (data not shown). Overall, these results indicate that the Δ*pstB2* mutant is severely limited in the phosphorus-containing compounds it can catabolize to meet its metabolic needs.

We also screened the OG1RF, Δ*pstB1*, and Δ*pstB2 phoZ* deletion strains on the PM4A plates in order to determine how removal of the AP gene would impact which phosphorus-containing molecules these strains were capable of catabolizing (Fig. 8). The OG1RFΔ*phoZ* and Δ*pstB1*Δ*phoZ* mutants were unable to grow in as many phosphate-containing compounds as the parent strains. This result suggests that the compounds which supported growth of AP-containing strains but not AP-deficient strains may be processed by AP outside of the cell prior to uptake through the Pst-PhoU importer. Like the Δ*pstB2* parent strain, the Δ*pstB2*Δ*phoZ* strain was incapable of substantial growth in CDM supplemented with any of the phosphorus-containing sources present on the PM4A plate.

Finally, we evaluated whether polyphosphate or the phosphonates APP, APB, AEP, and MPP could support growth of the Δ*pstB2* strain in CDM lacking all other phosphorus compounds. Polyphosphates are chains of Pi molecules, sometimes hundreds of molecules long, linked by energy-rich phosphoanhydride bonds (50). Phosphonates, which contain C−PO(OR)_2_ groups (R = alkyl, aryl, or H), are used by some bacteria as their sole sources of phosphorus (51), and *E. faecalis* has been shown to take them up (52). In addition, phosphonates have been isolated from the brains of cattle, which is one of the components used to make BHI (53). Neither OG1RF, Δ*pstB1*, nor Δ*pstB2* was able to grow in CDM supplemented with polyphosphate, APP, AEP, APB, or MPP as the sole phosphorus source (Fig. S6).

## DISCUSSION

Phosphorus is essential for the synthesis of many organic molecules vital for life, including ATP, phospholipids, and nucleic acids. Bacteria can acquire phosphorus from the environment in the form of Pi directly through specific importers, including the low-affinity, high-velocity Pit and the high-affinity, low-velocity Pst transporters (3). The coding region of the Pst transporter, the *pst-phoU* operon, has been well characterized in *E. coli* since it was initially described in the 1970s (3, 4, 54). Since then, the locus has been associated with adherence, fimbriae production, colonization, virulence, immune evasion, and antimicrobial resistance in *E. coli* and other gram-negative bacterial species (6, 7, 55–62). In gram-positive bacteria, where significantly less work characterizing *pst-phoU* loci has been published, disruption of normal expression of the *pst-phoU* locus has been shown to induce nutritional immunity in *Staphylococcus aureus* and reduce *Streptococcus mutans* adhesion to abiotic surfaces (63, 64). In *E. faecalis*, mutations in genes of the *pst-phoU* locus have been identified in several genetic studies. Specifically, mutations in *pstB1*, *pstB2*, and *pstC* were identified in clones that were serially passaged in pH 9 medium (65). Mutations in *pstB2* and *pstC* that were associated with small colony morphologies were also isolated from serially passaged biofilms grown in pH 9 medium (65). A *pstB2* mutation co-occurred with mutations in genes encoding a hypothetical protein and *N*-acetylmuramoyl-L-amidase in an *E. faecalis* clone isolated from *in vitro* evolution of a strain lacking the *croRS* two-component signaling system-encoding genes; the isolated clone grew faster than and had altered susceptibilities to vancomycin and teixobactin compared to the parental *croRS* deletion strain (66). Finally, transposon insertions in *phoU* have been identified in screens for genes needed for growth in nutrient-rich medium and biofilm formation (67, 68). Yet, despite this collection of genetic evidence that suggests broad roles for the *pst-phoU* locus genes in *E. faecalis* physiology, no follow-up functional characterization studies have been reported. In this work, we report the first direct investigation of any genes within the *E. faecalis pst-phoU* operon.

Many bacteria possess multiple homologs of genes found in the canonical *pst-phoU* operon in distinct chromosomal locations (Fig. 1). A unique and unstudied feature of the *pst-phoU* locus found in some Firmicutes, including *E. faecalis*, is the tandem arrangement of two ATPase-encoding *pstB* homologs (8, 69). Our findings demonstrate that the adjacent and non-identical *pstB1* and *pstB2* genes in the *pst-phoU* operon of *E. faecalis* OG1RF (Fig. 2) are differentially required for growth, phosphate uptake, and AP activation (Fig. 3, 5, and 6). Overexpression of *pstB2* in the Δ*pstB1* strain is sufficient to restore growth in low-Pi CDM (Fig. 2), phosphate uptake (Fig. 3), and *phoZ* expression with corresponding AP activity (Fig. 6) to wild-type levels. In contrast, overexpression of *pstB1* in the Δ*pstB2* strain does not rescue any of the phenotypes we evaluated, suggesting that *pstB2* is necessary for Pi uptake in *E. faecalis*.

Our data revealed that there is a significant change in expression of *pstB1* or *pstB2* when the tandem *pstB* homolog is deleted, and *in trans* overexpression of *pstB2* in the *pstB2* chromosomal deletion strain reduced the expression of *pstB1* (Fig. 4). The same strain [Δ*pstB2*(pPLK2-*pstB2*)] also took up Pi at a level similar to the Δ*pstB1* mutant, which was reduced relative to the wild-type strain (Fig. 5). We hypothesize that the decreased *pstB1* expression in the Δ*pstB2* complementation strain resulted in the strain phenocopying the Δ*pstB1* mutant in the Pi uptake assay. Data from the filamentous cyanobacterium *Nostoc punctiforme* provide evidence for regulatory interactions between *pstB* paralogs (34). To the best of our knowledge, *N. punctiforme* is the only microbe in which the effect of having multiple *pstB* genes has been investigated. *N. punctiforme* possesses four *pstB* genes (*pstB1–pstB4*) that are arranged across three distinct loci; *pstB1* and *pstB4* are in single copy in their respective loci that each also contain single copies of *pstS*, *pstC*, and *pstA*, while *pstB2* and *pstB3* are arranged in tandem in a third locus that has single copies of *pstC* and *pstA* and lacks *pstS* (34). Hudek et al. generated a Δ*pstB1* mutant and found that starving the strain of Pi led to increased expression of *pstB2* and *pstB4*, while *pstB3* expression remained the same when compared to the wild-type strain (34). Interestingly, they found that overexpression of *pstB1* led to reduced expression of *pstB3* compared to the wild-type strain following Pi starvation (34). However, the fact that *N. punctiforme* has four *pstB* paralogs and that Hudek et al. did not genetically disrupt the two copies that are tandemly arranged (*pstB2* and *pstB3*) makes it difficult to extrapolate the relevance of these findings to *E. faecalis*.

The Pho regulon consists of multiple genes and operons involved in the regulation of phosphate uptake and storage (70). Mutations within the *pst-phoU* operon are a known cause of *pho* regulon dysregulation, which results in increased AP activity (6, 33, 54, 71, 72). We found that deletion of either *pstB1* or *pstB2* results in increased AP-based enzymatic activity compared to wild-type, with disruption of *pstB2* having the highest levels of AP activity (Fig. 6). Additional experimentation will be necessary to determine the overall effect of deletion of *pstB1* or *pstB2* on Pho regulon activation in *E. faecalis*. The high AP activity of the Δ*pstB2* strain was largely responsible for the increased accumulation of extracellular Pi that we observed unexpectedly in the malachite green-based Pi uptake assays (Fig. 5 and 7). Despite this, there was still Pi accumulation in the medium from the Δ*pstB2*Δ*phoZ* sample (Fig. 7). One possible explanation for this observation is that *E. faecalis* may produce an as-yet-unidentified alkaline or acidic phosphatase that is capable of cleaving Pi from waste molecules. However, this explanation is unlikely given that none of the Δ*phoZ* strains cleaved the chromogenic substrate XP (Fig. 7A). An alternative explanation is that intracellular Pi is either leaked or actively exported from the cells into the surrounding medium. There are a number of known systems in bacteria that function through the export of Pi. For example, the systems encoded by the *glp*, *pgt*, and *uhp* loci are Pi antiporters that use the change in electrochemical gradient to import various organic phosphate molecules (73–75). Additionally, the *yjbB* gene encodes a Pi exporter, which is hypothesized to help maintain cellular phosphate homeostasis through the active export of Pi into the surrounding medium (76). Further studies will be necessary to determine if any such systems are present in the genome of *E. faecalis*.

We found that *pstB2* is dispensable for growth in BHI, a rich undefined medium, but is required for growth in medium in which Pi is the only source of phosphorus (Fig. 3). Supplementing phosphorus-free CDM with single alternate inorganic and organic phosphorus-containing compounds did not rescue growth of Δ*pstB2* (Fig. 8; Fig. S6), suggesting that deletion of *pstB2* results in a mutant that is incapable of taking up phosphorus efficiently from any single phosphorus-containing source. BHI and other rich undefined media likely contain large concentrations of multiple different phosphorus-containing molecules. Our data suggest that the Δ*pstB2* strain may require multiple phosphorus-containing molecules, as would be found in BHI, to meet its metabolic requirements. Our results also argue against *E. faecalis* OG1RF having a second functional dedicated Pi importer, such as the low-affinity, high-velocity Pit system that is present in *E. coli* (3, 4). Indeed, a BLAST search of the *E. faecalis* OG1RF genome (NCBI nucleotide database accession number: CP025020) for homologs of PitA or Pit B from *E. coli* strain W3110-P (NCBI nucleotide database accession number: NZ_CP084899) yielded only a single result: locus tag CVT43_09860 (OG1RF_11873), listed as encoding an “inorganic phosphate importer,” which was 32% and 29% identical to the amino acid sequences of PitA and PitB, respectively. If the CVT43_09860 (OG1RF_11873) locus does encode a Pit ortholog in *E. faecalis*, and it is functional, then it appears that its ability to take up Pi is insufficient to sustain growth on its own.

In conclusion, the results of this study support the hypothesis that *pstB2* encodes an ATPase that is required for Pi import in *E. faecalis*, while the ATPase encoded by *pstB1* has an accessory role in Pi import that can be duplicated when excess PstB2 is available. The data establish that *E. faecalis* is dependent on the *pst-phoU* operon to meet its Pi importation requirements. Therefore, targeting the Pst importer may be an effective strategy for future therapeutic interventions to combat antimicrobial-resistant enterococcal infections.

## SUPPLEMENTAL MATERIAL

Supplemental tables and figures jb.00033-25-s0001.docx Tables S1 to S5 and Figures S1 to S6.

ASM does not own the copyrights to Supplemental Material that may be linked to, or accessed through, an article. The authors have granted ASM a non-exclusive, world-wide license to publish the Supplemental Material files. Please contact the corresponding author directly for reuse.
