# Supplementary material for: The adjacent ATP-binding protein-encoding genes of the Enterococcus faecalis phosphate-specific transport (pst) locus have non-overlapping cellular functions
Source: J Bacteriol. 2025 Apr 14;207(5):e00033-25. doi: 10.1128/jb.00033-25 (PMC12096839; doi:10.1128/jb.00033-25)
Supplement: Supplemental tables and figures — Tables S1 to S5 and Figures S1 to S6. [file jb.00033-25-s0001.docx]

**Supplementary information**

# The adjacent ATP-binding protein-encoding genes of the *Enterococcus faecalis* phosphate specific transport (*pst*) locus have non-overlapping cellular functions

Christopher M. Healy, Evelyn A. Pham, Keane J. Dye, Candace N. Rouchon,

Biko McMillan, and Kristi L. Frank

Table S1. Strains analyzed in Figure 1.

| Species | Strain | Accession Number | Locus tag of first gene in operon |
| --- | --- | --- | --- |
| *Escherichia coli* | K-12 W3110-P | NZ_CP084899 | *pstS*: LHV60-RS18285 |
| *Vibrio cholerae* | O395 | Chromosome I: CP000627.1  Chromosome II: CP001236.1 | *pstC-1*: VC0395_A0258  *pstS:* VC395_A0063 |
| *Shigella sonnei* | Ss046 | CP000038.1 | *pstS*: SSON_3906 |
| *Shigella dysenteriae* | E670/74 | NZ_CP027027.1 | *pstS*: C5S64_RS07270 |
| *Pseudomonas aeruginosa* | PAO1-VE2 | NC_022591.1 | *pstS*: N296_RS27710 |
| *Bacillus anthracis* | A16 | NZ_CP001970.2 | First operon, *phoX*: A16_RS21875  Second operon, *pstO*: A16_RS03870 |
| *Staphylococcus aureus* | MRSA252 | BX571856 | SAR1402 |
| *Enterococcus faecalis* | OG1RF | CP025020 | *pstS2*: CVT43_07755 (OG1RF_11470)  *pstS:* CV43_07480 (OG1RF_11416) |
| *Streptococcus pyogenes* | M1 GAS | AE004092.2 | Spy_1245 |
| *Streptococcus pneumoniae* | D39 | NC_008533.2 | First operon, *pstS*: SPD_RS06585  Second operon: SPD_RS10125 |
| *Clostridioides difficile* | 630 | CP010905.2 | *pstS*: CDIF630_03568  *pstC*: CDIF630_03561 |

Table S2. *Enterococcus faecalis* strains used in this work.

| **Strain** | **Description** | **Reference** |
| --- | --- | --- |
| OG1RF | Wild-type Strain | [1] |
| OG1RF Δ*pstB1* | In-frame markerless deletion of *pstB1* | This study |
| OG1RF Δ*pstB2* | In-frame markerless deletion of *pstB2* | This study |
| OG1RF(pPLK2) | Wild-type strain with empty vector plasmid | This study |
| OG1RF Δ*pstB1*(pPLK2) | In-frame markerless deletion of *pstB1* with empty vector plasmid | This study |
| OG1RF Δ*pstB2*(pPLK2) | In-frame markerless deletion of *pstB2* with empty vector plasmid | This study |
| OG1RF(pPLK2-*pstB1*) | Wild-type strain with plasmid containing a wild-type copy of *pstB1* expressed *in trans* under control of the p23 promoter; overexpression strain | This study |
| OG1RF Δ*pstB1*(pPLK2-*pstB1*) | OG1RF Δ*pstB1* with wild-type copy of *pstB1* expressed *in trans* under control of the p23 promoter; complementation strain | This study |
| OG1RF Δ*pstB2*(pPLK2-*pstB1*) | OG1RF Δ*pstB2* with wild-type copy of *pstB1* expressed *in trans* under control of the p23 promoter; cross-complementation strain | This study |
| OG1RF(pPLK2-*pstB2*) | Wild-type strain with plasmid containing a wild-type copy of *pstB2* expressed *in trans* under control of the p23 promoter; overexpression strain | This study |
| OG1RF Δ*pstB1*(pPLK2-*pstB2*) | OG1RF Δ*pstB1* with wild-type copy of *pstB2* expressed *in trans* under control of the p23 promoter; cross-complementation strain | This study |
| OG1RF Δ*pstB2*(pPLK2-*pstB2*) | OG1RF Δ*pstB2* with wild-type copy of *pstB2* expressed *in trans* under control of the p23 promoter; complementation strain | This study |
| OG1RF Δ*phoZ*(pPLK2) | In-frame markerless deletion of *phoZ* with empty vector plasmid | This study |
| OG1RF Δ*pstB1*Δ*phoZ*(pPLK2) | In-frame markerless deletion of *phoZ* in the OG1RF Δ*pstB1* parental strain, contains empty vector plasmid | This study |
| OG1RF Δ*pstB2*Δ*phoZ*(pPLK2) | In-frame markerless deletion of *phoZ* in the OG1RF Δ*pstB2* parental strain, contains empty vector plasmid | This study |
| OG1RF Δ*phoZ* (pPLK2-*phoZ*) | In-frame markerless deletion of *phoZ* with wild-type copy of *phoZ* expressed *in trans* under control of the p23 promoter | This study |
| OG1RF Δ*pstB1*Δ*phoZ*(pPLK2-*phoZ*) | In-frame markerless deletion of *phoZ* in the OG1RF Δ*pstB1* parental strain with wild-type copy of *phoZ* expressed *in trans* under control of the p23 promoter | This study |
| OG1RF Δ*pstB2*Δ*phoZ*(pPLK2-*phoZ*) | In-frame markerless deletion of *phoZ* in the Δ*pstB2* parental strain with wild-type copy of *phoZ* expressed *in trans* under control of the p23 promoter | This study |

Table S3. Plasmids used in this work.

| **Plasmid** | **Description** | **Reference** |
| --- | --- | --- |
| pCJK218 | Allelic exchange vector; confers chloramphenicol resistance | [2] |
| pCJK47 | Allelic exchange vector; confers erythromycin resistance | [3] |
| pPLK2 | Complementation vector; contains the constitutive p23 promoter from *Lactococcus lactis*; confers chloramphenicol resistance | [4] |
| pPLK2-*pstB1* | pPLK2 with wild-type copy of *pstB1* and native RBS expressed from p23 promoter | This study |
| pPLK2-*pstB2* | pPLK2 with wild-type copy of *pstB2* and native RBS expressed from p23 promoter | This study |
| pPLK2-*phoZ* | pPLK2 with wild-type copy of *phoZ* and native RBS expressed from p23 promoter | This study |

Table S4. Oligonucleotides used in this work.

| **Primer Name** | **Sequence (5’-3’)** | **Reference** |
| --- | --- | --- |
| OG11471-*pstS2* For | GTTGTACCAACAAATTGATAAC | This study |
| OG11471-*pstS2* Rev | CAAAGTAAGTATACTTGCCAA | This study |
| *pstS*2-*pstC* For | GGCAAGGAAATGTCATTAA | This study |
| *pstS*2-*pstC* Rev | AATTTTTTCTGTACATCTTCC | This study |
| *pstC*-*pstA* For | GATGTCTTTACTATTCAACAT | This study |
| *pstC*-*pstA* Rev | GCCAAAATTAAAACAATGAC | This study |
| *pstA*-*pstB2* For | GCAACCGTCTTTATAAGAG | This study |
| *pstA*-*pstB2* Rev | ACATGTAAGTCTTCCGT | This study |
| *pstB2*-*pstB1* For | GACCATACAAGAAAAATCTTTA | This study |
| *pstB2*-*pstB1* Rev | CATGGCAGTTAATTCTCCTT | This study |
| *pstB1*-*phoU* For | CATTTCTGGTAAATTTGGTTAA | This study |
| *pstB1*-*phoU* Rev | CATGATTAATATATGCCTTCAC | This study |
| *phoU*-*liaX* For | CCGAATTGAATAGTAATCGTG | This study |
| *phoU*-*liaX* Rev | CCTTGTTTTTGATCATTTGTC | This study |
| EF1755(pstB1)-upF | GGCTGTGCATATTTGGAAAATT | This study |
| EF1755(pstB1)-downR | CACTAATTCTAATACGCGTTCTC | This study |
| EF1755(pstB1)-2stepF | TTTGGTTAGGAGGAGCAAGATGGGCAAATTTGGTTA AAAGGGGGAAAAAGAATG | This study |
| EF1755(pstB1)-2stepR | CATTCTTTTTCCCCCTTTTAACCAAATTTGCCC ATCTTGCTCCTCCTAACCAAA | This study |
| *pstB1* Forward Cloning | GCCGTCTAGAGTTAGGAGGAGCAAGATGG | This study |
| *pstB1* Reverse Cloning | GCGCGCAAGCTTTTAACCAAATTTACCAGAAATG | This study |
| *pstB2*-upF_BamHI | ATCGGGATCCAAACAACGTTCTTTGGTCTTTA | This study |
| *pstB2*-downR_SphI | ATCGGCATGCGTTGTTGTAACGCAATCATTTC | This Study |
| *pstB2*-2stepF | CTTAATGTTAGGGGAATTAGCATGAAAGAATTTGGTTAGGAGGAGCAAGATGGGCAAAG | This study |
| *pstB2*-2stepR | CTTTGCCCATCTTGCTCCTCCTAACCAAATTCTTTCATGCTAAATCCCCTAACATTAAG | This study |
| *pstB1* Forward Cloning | CATTTCTGGTAAATTTGGTTAA | This study |
| *pstB1* Reverse Cloning | CATGATTAATATATGCCTTCAC | This study |
| *pstB2* Forward Cloning | GGCCGTCTAGAGTTAGGGGAATTAGCATG | This study |
| *pstB2* Reverse Cloning | GCGCGCAAGCTTCCCATCTTGCTCCTCCTA | This study |
| *phoZ* Forward Cloning | GGCGAGCTCTTATTTACCAATACCTTTATCTTTAAT | This study |
| *phoZ* Reverse Cloning | GGCGAGCTCTATGGGTAGATATGAAGGAG | This study |
| RT*pstB2*-F | CGTTTCTTCAACCGTTCCTG | This study |
| RT*pstB2*-R | AAGCGCCTTAGCTCTGTCTG | This study |
| RT*pstB1*-F | ACAAACCCGCTGTTGTTGTC | This study |
| RT*pstB1*-R | CTTGCGCCTAAAAGGAGTCA | This study |
| RT*phoU*-F | CATGGTCCGCCATTCTTT | This study |
| RT*phoU*-R | GCGTTACAACAACCAGTGAC | This study |
| *gyrB*-Forward | CAAGCCAAAACAGGTCGCC | [5] |
| *gyrB*-Reverse | ACCAACACCGTGCAAGCC | [5] |

| **Table S5. Statistical analysis for Figure 3, middle row: Growth curve of strains grown in 0.2 mM Pi CDM.^1^** | | | | | | | | | |
| --- | --- | --- | --- | --- | --- | --- | --- | --- | --- |
| **Time (hours)** | **OG1RF(pPLK2) vs. Δ*pstB1*(pPLK2)** | **OG1RF(pPLK2) vs. Δ*pstB2*(pPLK2)** | **Δ*pstB1*(pPLK2) vs. Δ*pstB2*(pPLK2)** | **OG1RF(pPLK2-*pstB1*) vs. Δ*pstB1*(pPLK2-*pstB1*)** | **OG1RF(pPLK2-*pstB1*) vs. Δ*pstB2*(pPLK2-*pstB1*)** | **Δ*pstB1*(pPLK2-*pstB1*)vs. Δ*pstB2*(pPLK2-*pstB1*)** | **OG1RF(pPLK2-*pstB2*) vs. Δ*pstB1*(pPLK2-*pstB2*)** | **OG1RF(pPLK2-*pstB2*) vs. Δ*pstB2*(pPLK2-*pstB2*)** | **Δ*pstB1*(pPLK2-*pstB2* )vs. Δ*pstB2*(pPLK2-*pstB2*)** |
| **0** |  |  |  |  |  |  |  |  |  |
| **0.5** |  |  |  |  |  |  |  |  |  |
| **1** |  |  |  |  |  |  |  |  |  |
| **1.5** |  |  |  |  |  |  |  |  |  |
| **2** |  |  |  |  |  |  |  |  |  |
| **2.5** |  |  |  |  |  |  |  |  |  |
| **3** |  |  |  |  |  |  |  |  |  |
| **3.5** |  |  |  |  |  |  |  |  |  |
| **4** |  |  |  |  |  | ** |  |  |  |
| **4.5** |  | ** |  |  | ** | **** |  |  |  |
| **5** | ** | **** |  |  | **** | **** |  |  |  |
| **5.5** | *** | **** |  |  | **** | **** |  |  |  |
| **6** | **** | **** | ** |  | **** | **** |  |  |  |
| **6.5** | **** | **** | *** |  | **** | **** |  |  |  |
| **7** | **** | **** | **** |  | **** | **** |  |  |  |
| **7.5** | **** | **** | **** |  | **** | **** |  |  |  |
| **8** | **** | **** | **** |  | **** | **** |  |  |  |
| **8.5** | **** | **** | **** |  | **** | **** |  |  |  |
| **9** | **** | **** | **** |  | **** | **** |  |  |  |
| **9.5** | ** | **** | **** | ** | **** | **** |  |  |  |
| **10** | ** | **** | **** | **** | **** | **** |  |  |  |
| **10.5** | ** | **** | **** | **** | **** | **** |  |  |  |
| **11** | ** | **** | **** | **** | **** | **** |  |  |  |
| **11.5** | ** | **** | **** | **** | **** | **** |  |  |  |
| **12** | ** | **** | **** | **** | **** | **** |  |  |  |
| **12.5** | ** | **** | **** | **** | **** | **** |  |  |  |
| **13** | *** | **** | **** | **** | **** | **** |  |  |  |
| **13.5** | *** | **** | **** | **** | **** | **** |  |  |  |
| **14** | ** | **** | **** | **** | **** | **** |  |  |  |
| **14.5** | ** | **** | **** | **** | **** | **** |  |  |  |
| **15** | ** | **** | **** | **** | **** | **** |  |  |  |
| **15.5** | ** | **** | **** | **** | **** | **** |  |  |  |
| **16** | ** | **** | **** | **** | **** | **** |  |  |  |

^1^Two-way ANOVA with Tukey’s correction: **, p <0.01; ***, p <0.001; ****, p <0.0001.

**Table S6. Statistical analysis for Figure 3, bottom row: Growth curve of strains grown in 80 mM Pi CDM.^1^**

| **Time (hours)** | **OG1RF(pPLK2) vs. Δ*pstB1*(pPLK2)** | **OG1RF(pPLK2) vs. Δ*pstB2*(pPLK2)** | **Δ*pstB1*(pPLK2) vs. Δ*pstB2*(pPLK2)** | **OG1RF(pPLK2-*pstB1*) vs. Δ*pstB1*(pPLK2-*pstB1*)** | **OG1RF(pPLK2-*pstB1*) vs. Δ*pstB2*(pPLK2-*pstB1*)** | **Δ*pstB1*(pPLK2-*pstB1*) vs. Δ*pstB2*(pPLK2-*pstB1*)** | **OG1RF(pPLK2-*pstB2*) vs. Δ*pstB1*(pPLK2-*pstB2*)** | **OG1RF(pPLK2-*pstB2*) vs. Δ*pstB2*(pPLK2-*pstB2*)** | **Δ*pstB1*(pPLK2-*pstB2*) vs. Δ*pstB2*(pPLK2-*pstB2*)** |
| --- | --- | --- | --- | --- | --- | --- | --- | --- | --- |
| **0** |  |  |  |  |  |  |  |  |  |
| **0.5** |  |  |  |  |  |  |  |  |  |
| **1** |  |  |  |  |  |  |  |  |  |
| **1.5** |  |  |  |  |  |  |  |  |  |
| **2** |  |  |  |  |  |  |  |  |  |
| **2.5** |  |  |  |  |  |  |  |  |  |
| **3** |  |  |  |  |  |  |  |  |  |
| **3.5** |  |  |  |  |  |  |  |  |  |
| **4** |  |  |  |  |  |  |  |  |  |
| **4.5** |  | * |  |  |  |  |  |  |  |
| **5** |  | *** | * |  |  |  |  |  |  |
| **5.5** | ** | **** | *** |  |  |  |  |  |  |
| **6** | **** | **** | **** |  | ** | ** |  | * |  |
| **6.5** | **** | **** | **** |  | **** | **** | * | ** |  |
| **7** | **** | **** | **** |  | **** | **** | * | * |  |
| **7.5** | **** | **** | **** |  | **** | **** | * |  |  |
| **8** | **** | **** | **** |  | **** | **** |  |  |  |
| **8.5** | **** | **** | **** |  | **** | **** |  |  | * |
| **9** | **** | **** | **** |  | **** | **** |  |  | ** |
| **9.5** | **** | **** | **** |  | **** | **** | * |  | **** |
| **10** | **** | **** | **** |  | **** | **** | *** |  | **** |
| **10.5** | **** | **** | **** |  | **** | **** |  |  |  |
| **11** | **** | **** | **** |  | **** | **** |  |  |  |
| **11.5** |  | **** | **** |  | **** | **** |  |  |  |
| **12** |  | **** | **** |  | **** | **** |  |  |  |
| **12.5** |  | **** | **** |  | **** | **** |  |  |  |
| **13** |  | **** | **** |  | **** | **** |  |  |  |
| **13.5** |  | **** | **** |  | **** | **** |  |  |  |
| **14** |  | **** | **** |  | **** | **** |  |  |  |
| **14.5** | * | **** | **** |  | **** | **** |  |  |  |
| **15** | * | **** | **** |  | **** | **** |  |  |  |
| **15.5** | ** | **** | **** |  | **** | **** |  |  |  |
| **16** | ** | **** | **** |  | **** | **** |  |  |  |

^1^Two-way ANOVA with Tukey’s correction: *, p <0.05; **, p <0.01; ***, p <0.001; ****, p <0.0001


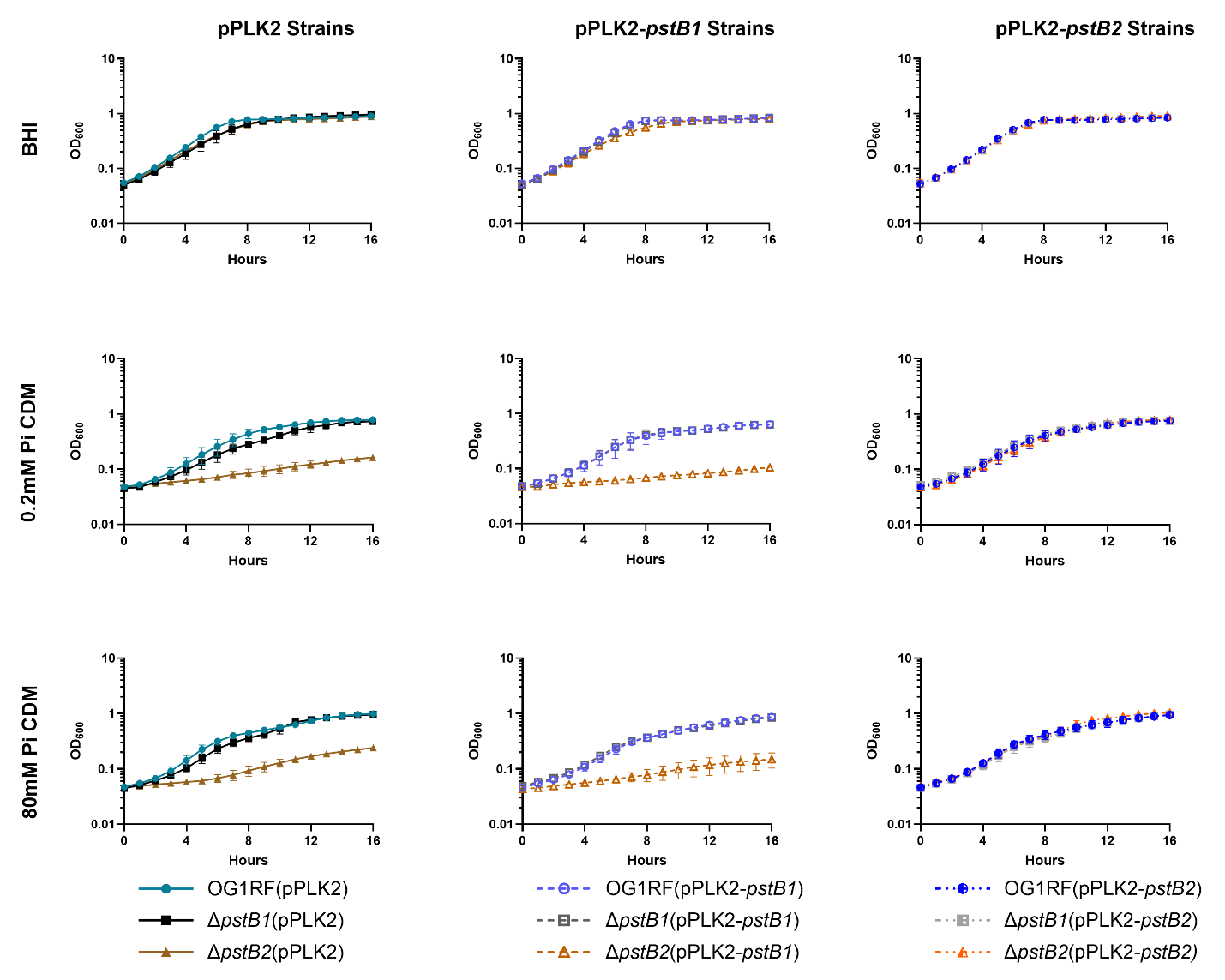


**Figure S1. Δ*pstB1* exhibits a growth defect in low Pi CDM, and CDM does not support growth of ΔpstB2 at 25°C.**

Bacterial growth at an incubation temperature of 25°C was monitored over time for strains grown in (top) BHI, (middle) CDM containing 0.2 mM Pi (low Pi CDM), and (bottom) CDM containing 80 mM Pi (high Pi CDM). Graphs in the left-most column show only strains that carry the empty vector (pPLK2). The second and third columns show strains that express *pstB1* or *pstB2*, respectively, *in trans* from pPLK2. Each data point is the mean of three biological replicates. Error bars show the standard deviation.


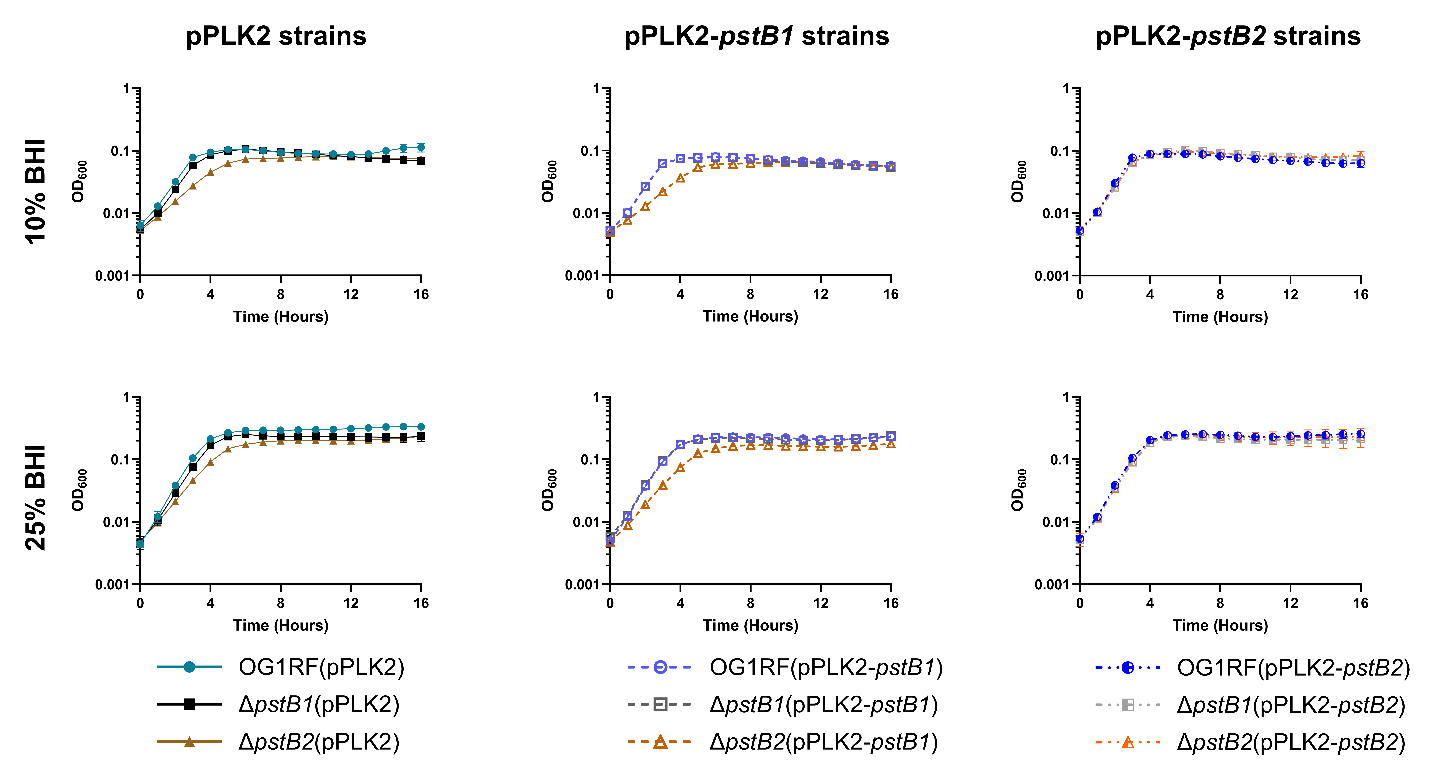


**Figure S2. Growth curves in 10% (top row) and 25% BHI (bottom row) at 37ºC.**

Bacterial strains were seeded at an OD_600 nm_ = 0.01 and incubated for 16 hours statically at 37ºC. Bacterial growth was monitored over time. Graphs in the left-most column show only strains that carry the empty vector (pPLK2). The second and third columns show strains that express *pstB1* or *pstB2*, respectively, *in trans* from pPLK2. Each data point is the mean of three biological replicates. Error bars show the standard deviation.


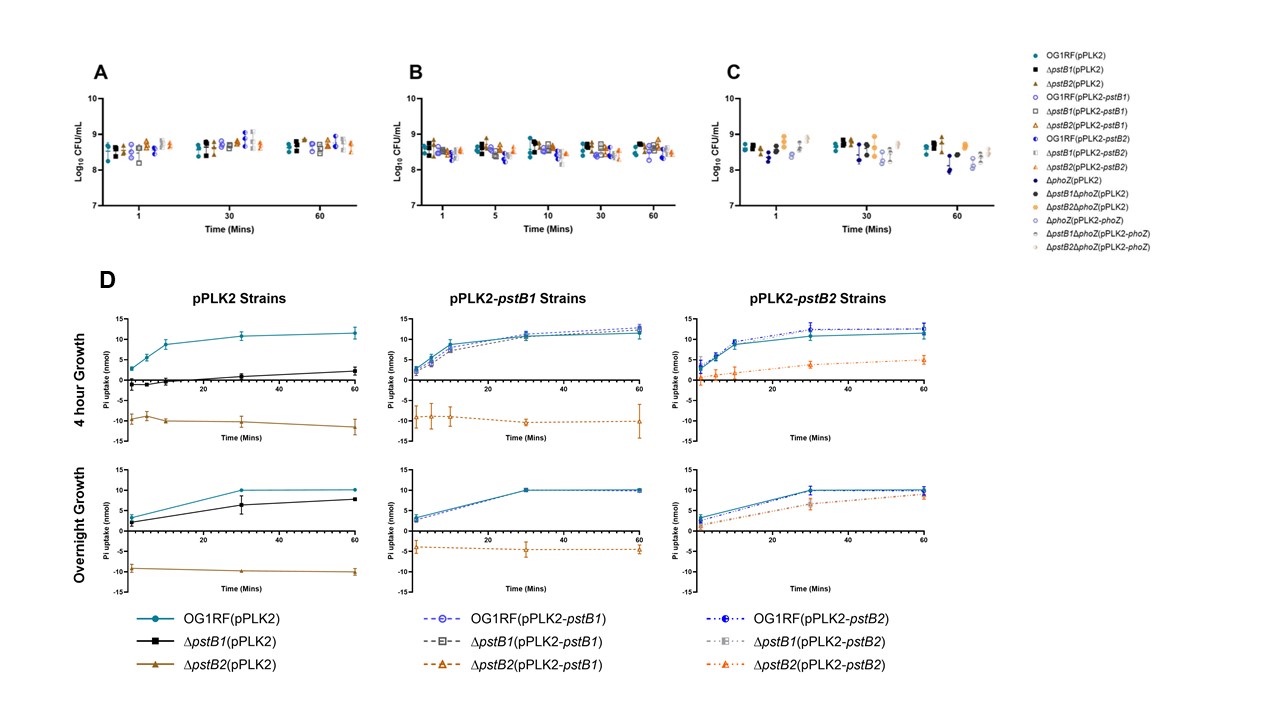


**Figure S3. Number of viable bacteria used in malachite green-based Pi uptake assays (A-C), and alternate representation of Figure 5 from the main text (D).** Following incubation for the indicated times, and prior to filtration, 100 µL of bacterial cells mixed with K_2_HPO_4_ were serially diluted and plated on BHI agar to enumerate the CFU/mL. Strains were either (A, B) wild-type for *phoZ* or (C) *phoZ* deletion mutants. Cells were grown for (A) 4 hours or (B, C) overnight in 25% BHI. Each data point shows a biological replicate. Horizontal lines and error bars indicate the mean and standard deviation, respectively. There were no significant differences among the CFU/ml counts in any panel. (D) Graphs in the left-most column show only strains that carry the empty vector (pPLK2). The second and third columns show strains that express *pstB1* or *pstB2*, respectively, *in trans* from pPLK2. See legend for Figure 5 in main text for additional details.


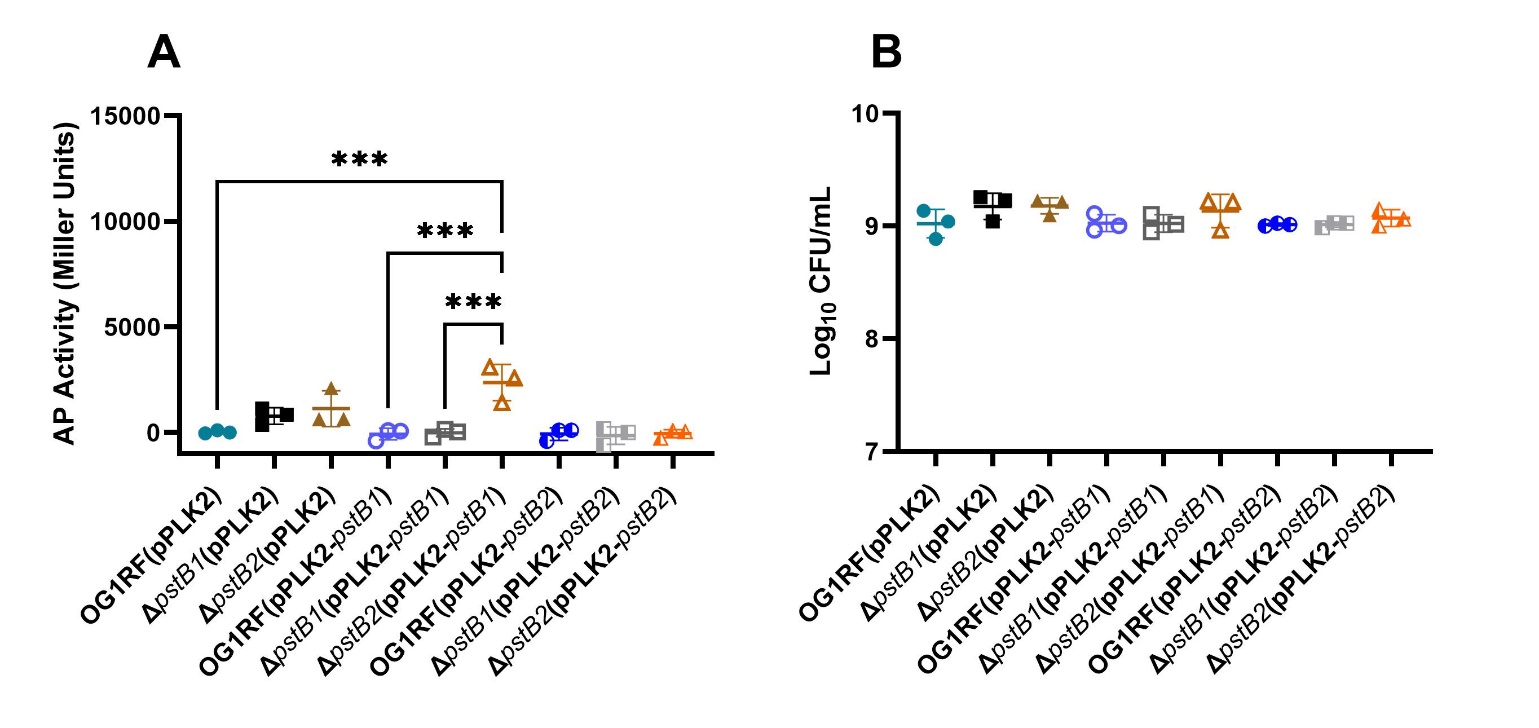


Figure S4. AP activity in supernatants taken from Δ*pstB1* and Δ*pstB2* cultures compared to the wild-type strain.

(A) Quantification of AP activity as measured by colorimetric assay of supernatants exposed to pNPP. Supernatants from cells grown to mid-log phase were incubated with 0.4% pNPP at 37°C for 10 mins, mixed with stop solution, and then the reactions were measured for color changes on a plate reader. One-way ANOVA with Tukey correction: ***, p <0.001. (B) Aliquots from each strain at mid-log phase were serial diluted and plated on BHI to enumerate CFU/ml. There were no significant differences in the CFU/ml counts between strains at each time point. Outliers were identified by application of the Grubb’s Test and excluded from the data set. For (A) and (B), each data point represents an independent biological replicate, and horizontal bars indicate the mean.


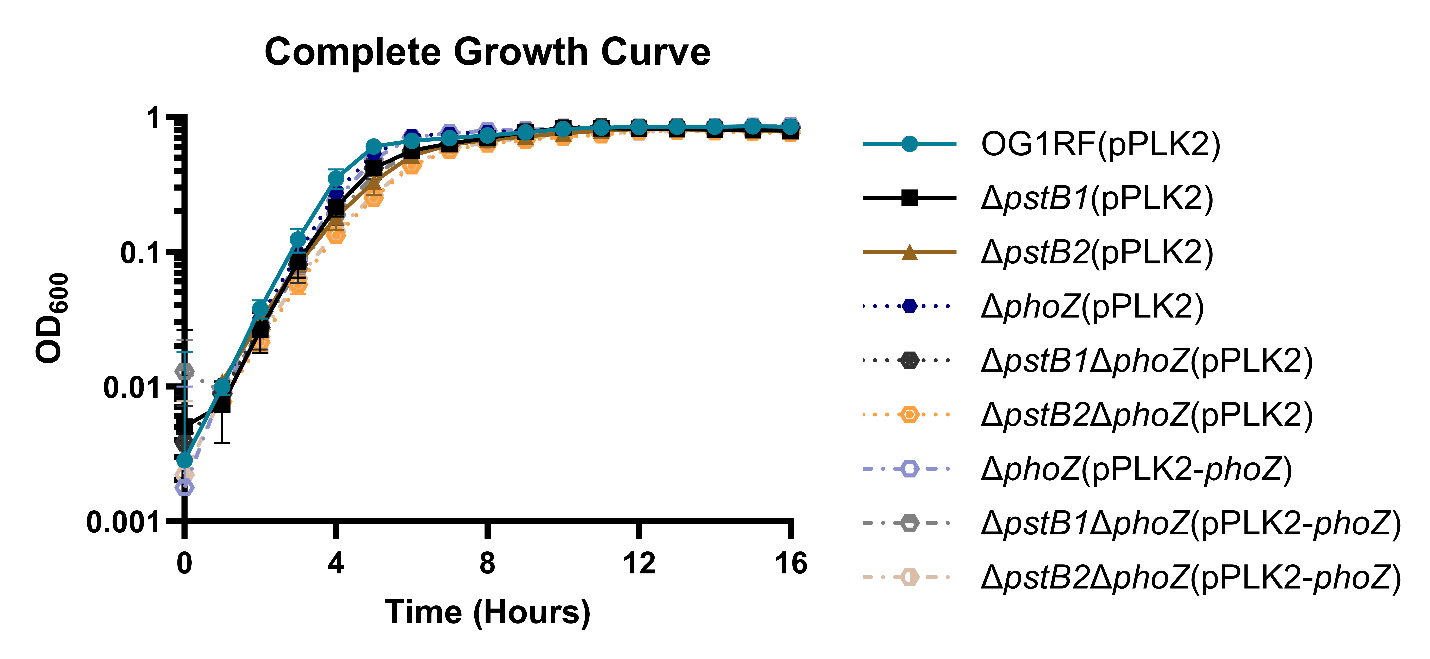


**Figure S5. Growth curves of Δ*phoZ* strains in BHI.**

Strains were seeded at an OD_600 nm_ = 0.01 in BHI and incubated for 16 hours statically at 37ºC. Bacterial growth was monitored over time. Each data point is the average of three biological replicates. Error bars show the standard deviation.


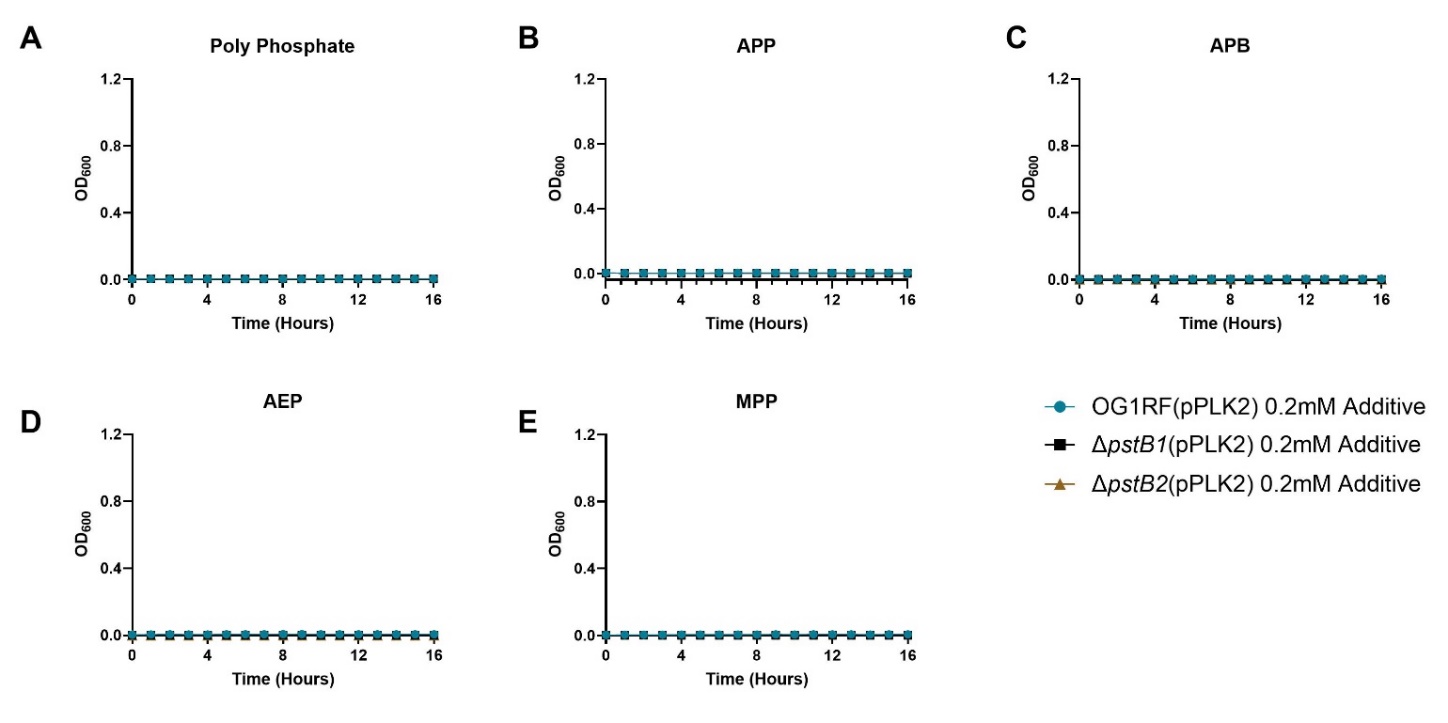


**Figure S6. Growth of *E. faecalis* strains in CDM supplemented with polyphosphate or various phosphonates.**

APP, DL-2-amino-3-phosphonopropionic acid; APB, (+/-)-2-amino-4-phosphonobutyric acid; AEP, 2-aminoethylphosphonic acid; MPP, methylphosphonic acid.

**REFERENCES**

1. **Dunny GM, Brown BL, Clewell DB**. Induced cell aggregation and mating in *Streptococcus faecalis*: evidence for a bacterial sex pheromone. *Proceedings of the National Academy of Sciences USA* 1978;75(7):3479-3483.

2. **Vesic D, Kristich CJ**. A Rex family transcriptional repressor influences H_2_O_2_ accumulation by *Enterococcus faecalis*. *J Bacteriol* 2013;195(8):1815-1824.

3. **Kristich CJ, Chandler JR, Dunny GM**. Development of a host-genotype-independent counterselectable marker and a high-frequency conjugative delivery system and their use in genetic analysis of *Enterococcus faecalis*. *Plasmid* 2007;57(2):131-144.

4. **Rouchon CN, Weinstein AJ, Hutchison CA, Zubair-Nizami ZB, Kohler PL, Frank KL**. Disruption of the *tagF* orthologue in the *epa* locus variable region of *Enterococcus faecalis* causes cell surface changes and suppresses an *eep*-dependent lysozyme resistance phenotype. *J Bacteriol* 2022;204(10):e0024722.

5. **Bourgogne A, Singh KV, Fox KA, Pflughoeft KJ, Murray BE, Garsin DA**. EbpR is important for biofilm formation by activating expression of the endocarditis and biofilm-associated pilus operon (*ebpABC*) of *Enterococcus faecalis* OG1RF. *J Bacteriol* 2007;189(17):6490-6493.
